# Supplementary material for: Aspirin mediates protection from diabetic kidney disease by inducing ferroptosis inhibition
Source: PLoS One. 2022 Dec 14;17(12):e0279010. doi: 10.1371/journal.pone.0279010 (PMC9749971; doi:10.1371/journal.pone.0279010)
Supplement: S1 Table — (DOCX) [file pone.0279010.s001.docx]

|  | Human | mice |
| --- | --- | --- |
| GPX4-F | CCCGATACGCTGAGTGTGGTTTG | ATAAGAACGGCTGCGTGGTGAAG |
| GPX4-R | TCTTCGTTACTCCCTGGCTCCTG | TAGAGATAGCACGGCAGGTCCTTC |
| FTH1-F | AGAACTACCACCAGGACTCAGAGG | CAGCGAGGTGGCCGAATCTTC |
| FTH1-R | AGCCACATCATCGCGGTCAAAG | AGCCAGTTTGTGCAGTTCCAGTAG |
| TFR1-F | TGAGGGAGGAGCCAGGAGAGG | CCCGTTGTTGAGGCAGACCTTG |
| TFR1-R | CTTGATGGTGCCGGTGAAGTCTG | CCTGATGACTGAGATGGCGGAAAC |
| PTGS2-F | AATCTGGCTGCGGGAACACAAC | GGTGCCTGGTCTGATGATGTATGC |
| PTGS2-R | TGTCTGGAACAACTGCTCATCACC | GGATGCTCCTGCTTGAGTATGTCG |
| SLC7A11-F | ACGGTGGTGTGTTTGCTGTCTC | ACCACCATCAGTGCGGAGGAG |
| SLC7A11-R | GCTGGTAGAGGAGTGTGCTTGC | ATGGAGCCGAAGCAGGAGAGG |
| Kim-1 F | GCCACTTCACCATCTTCACCTCAG | CCTGCTGCTACTGCTCCTTGTG |
| Kim-1 R | ACGGTGTCATTCCCATCTGTTGTG | CCACGCTTAGAGATGCTGACTTCC |
| NGAL F | GAACCAAGGAGCTGACTTCGGAAC | AGGGCTGTCGCTACTGGATCAG |
| NGAL R | GATTGGGACAGGGAAGACGATGTG | CGAACTGGTTGTAGTCCGTGGTG |
| hCOX2 sense（5'-3'） | CGUUGUGAAUAACAUUCCCUUTT |  |
| hCOX2 antisense（5'-3'） | AAGGGAAUGUUAUUCACAACGTT |  |
