# Supplementary material for: Aspirin mediates protection from diabetic kidney disease by inducing ferroptosis inhibition
Source: PLoS One. 2022 Dec 14;17(12):e0279010. doi: 10.1371/journal.pone.0279010 (PMC9749971; doi:10.1371/journal.pone.0279010)

Fig1C

Western blot analysis of GPX4, SLC7A11, FTH-1, COX2 and TFR-1 protein expression in cells in the Ctrl and HG groups

COX2

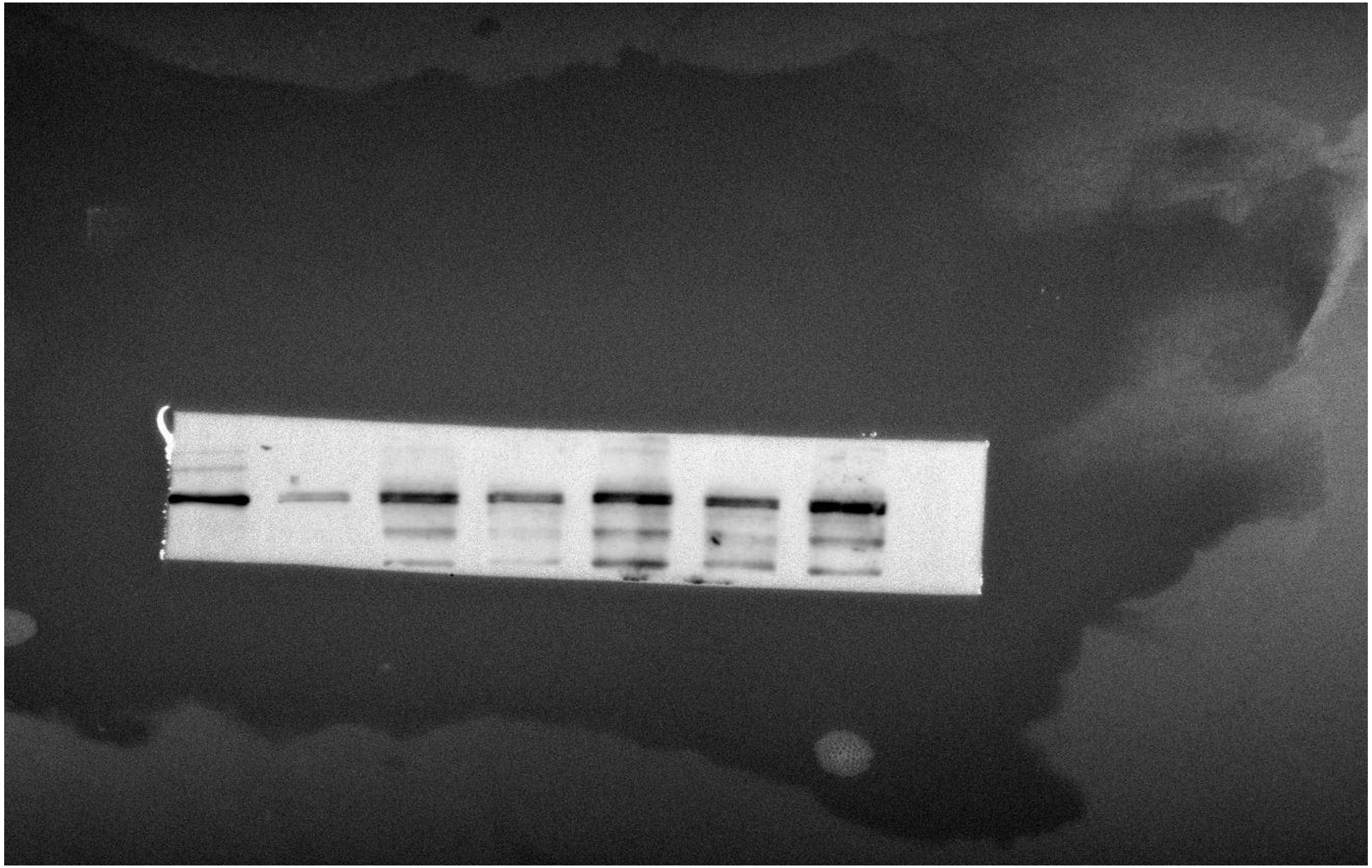

Fig1C

TFR-1

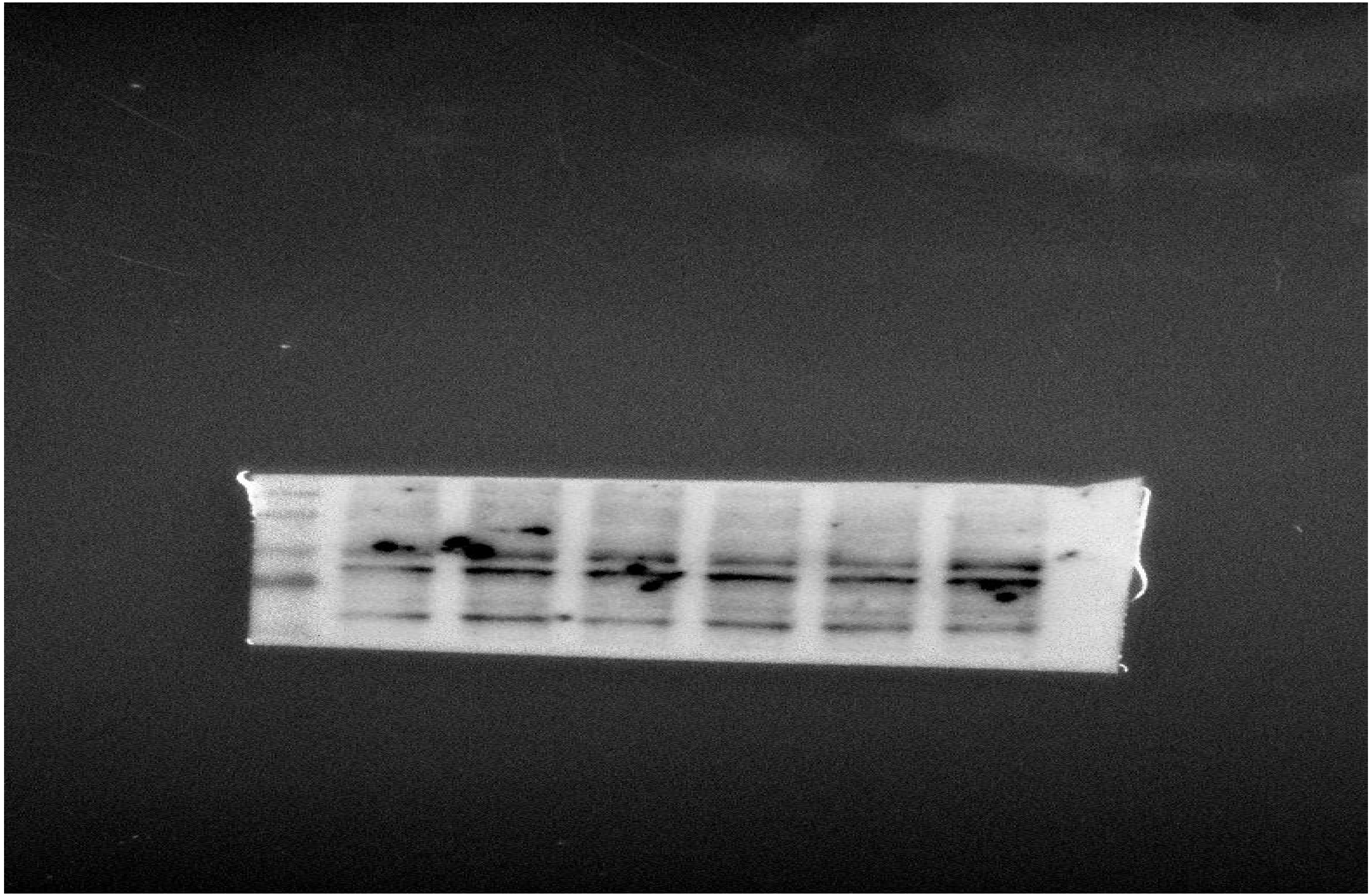

Fig1C

SLC7A11

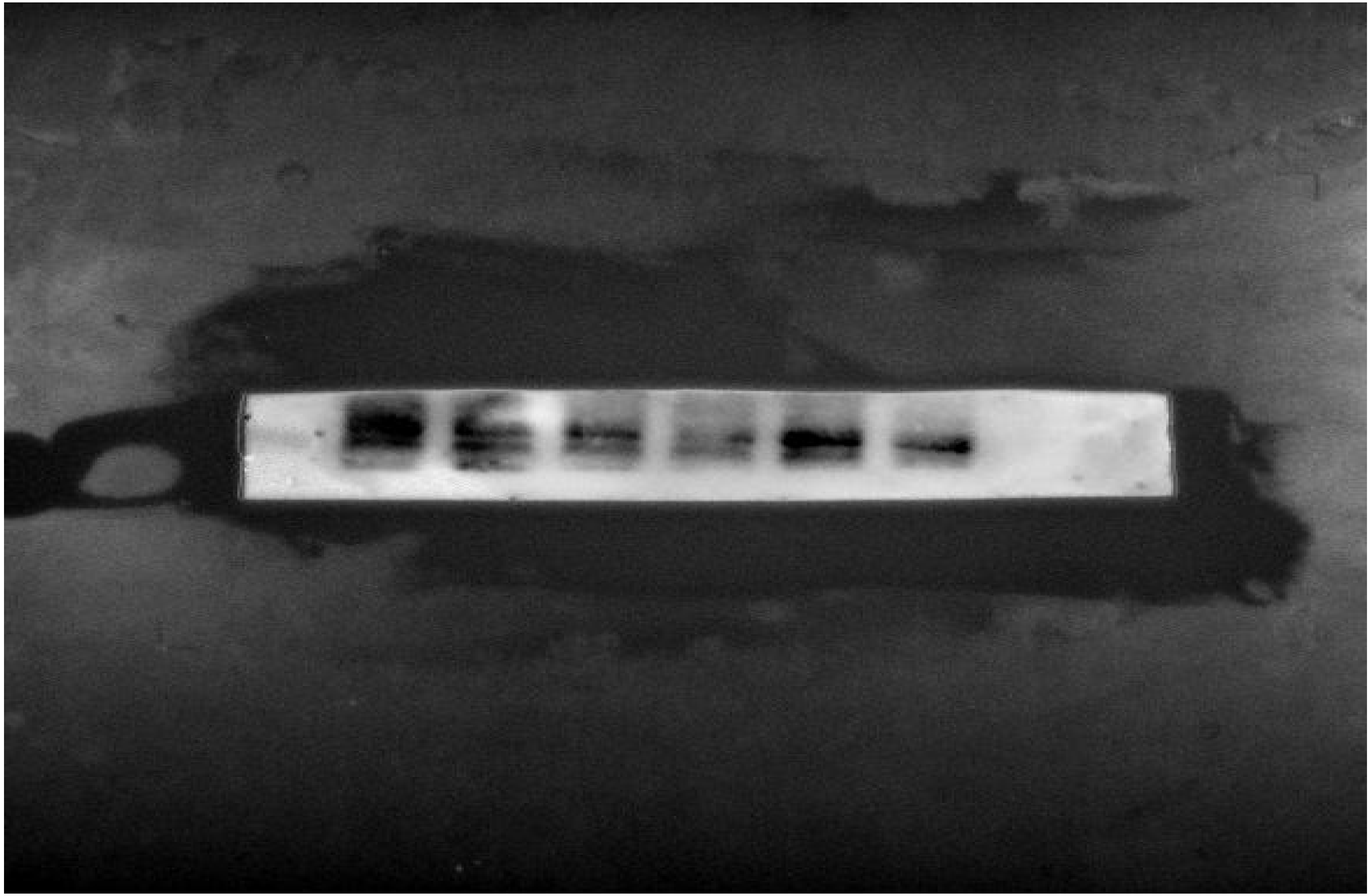

Fig1C

FTH-1

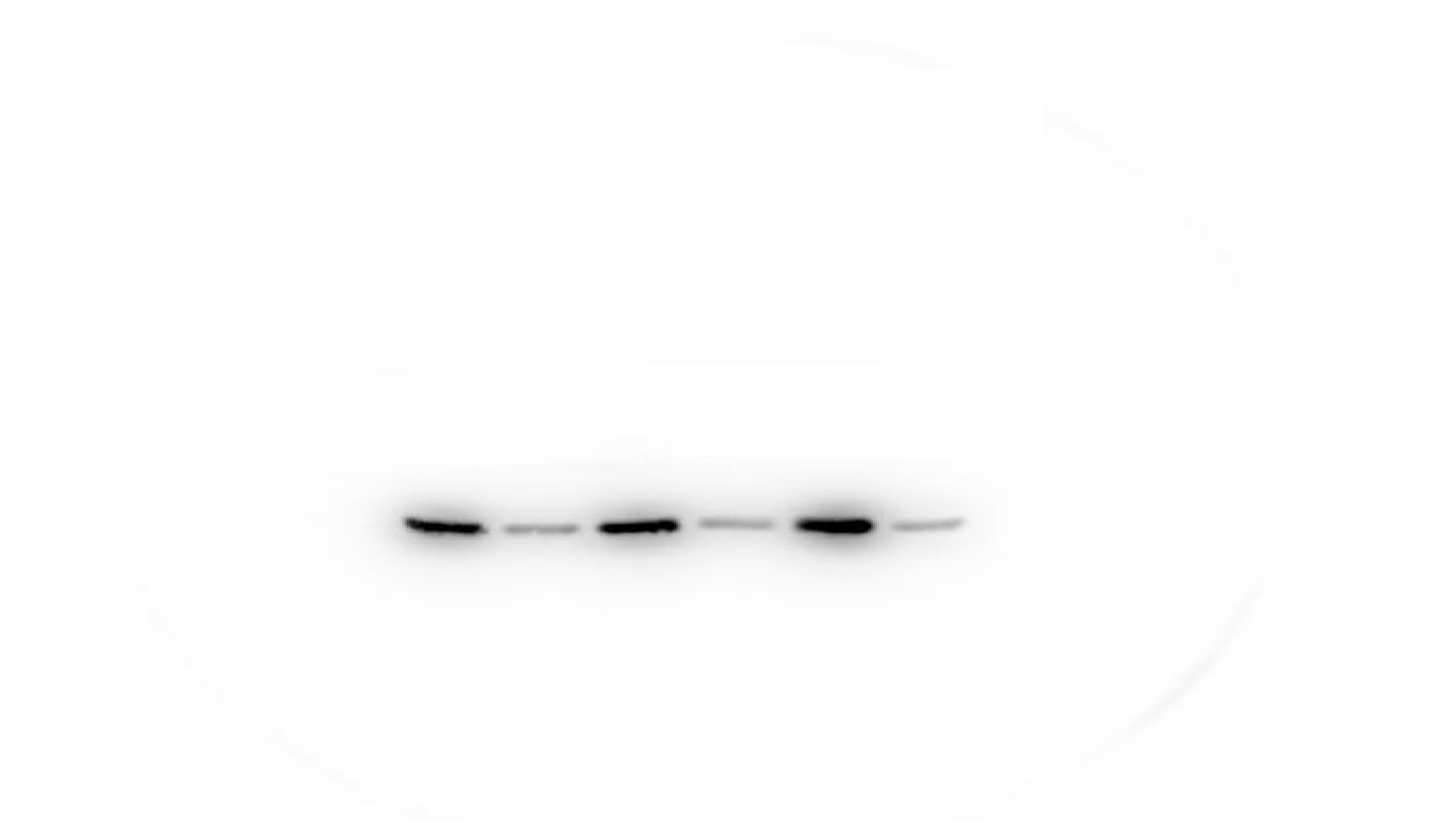

Fig1C

GPX4

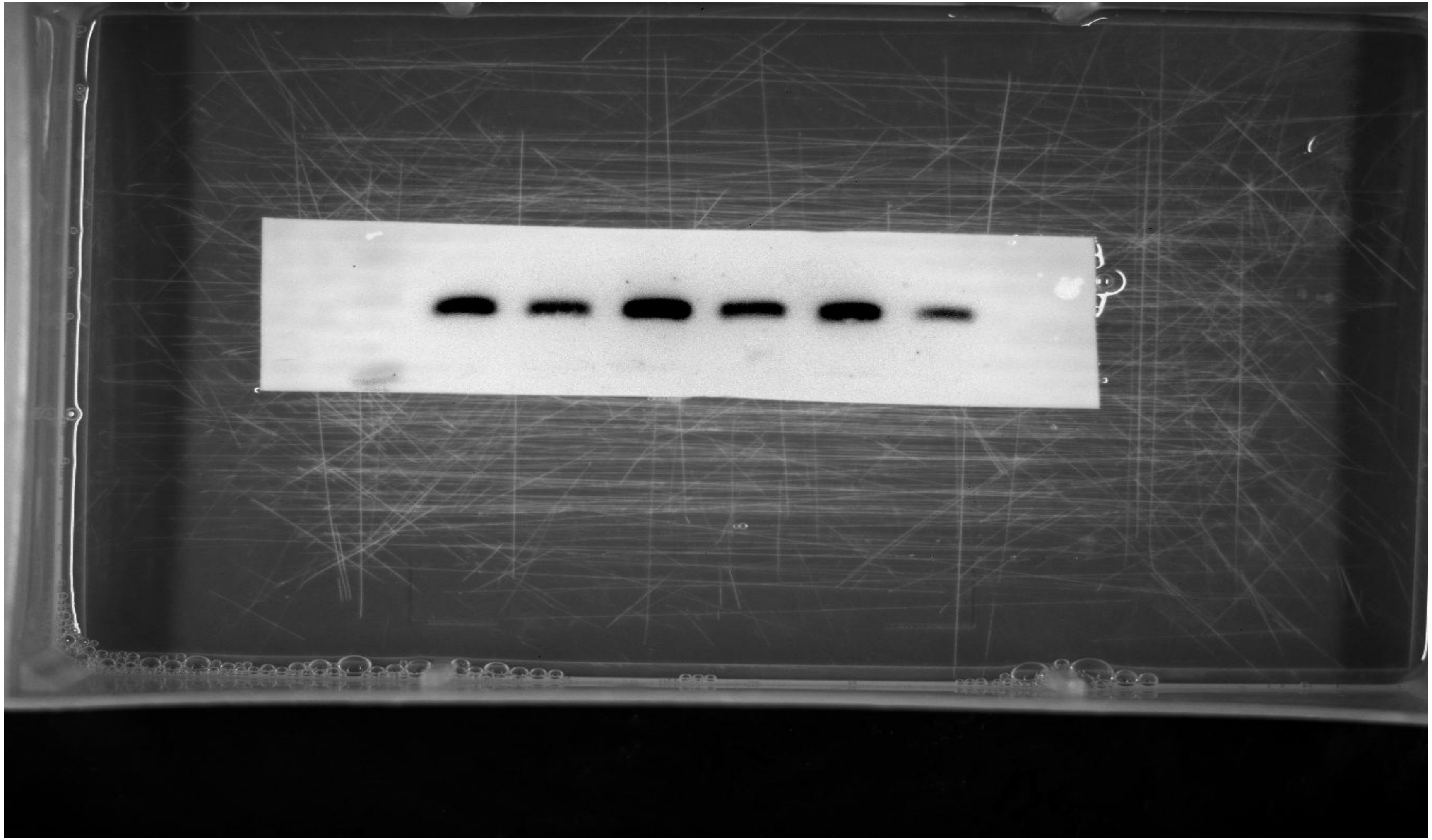

Fig1C

GAPDH

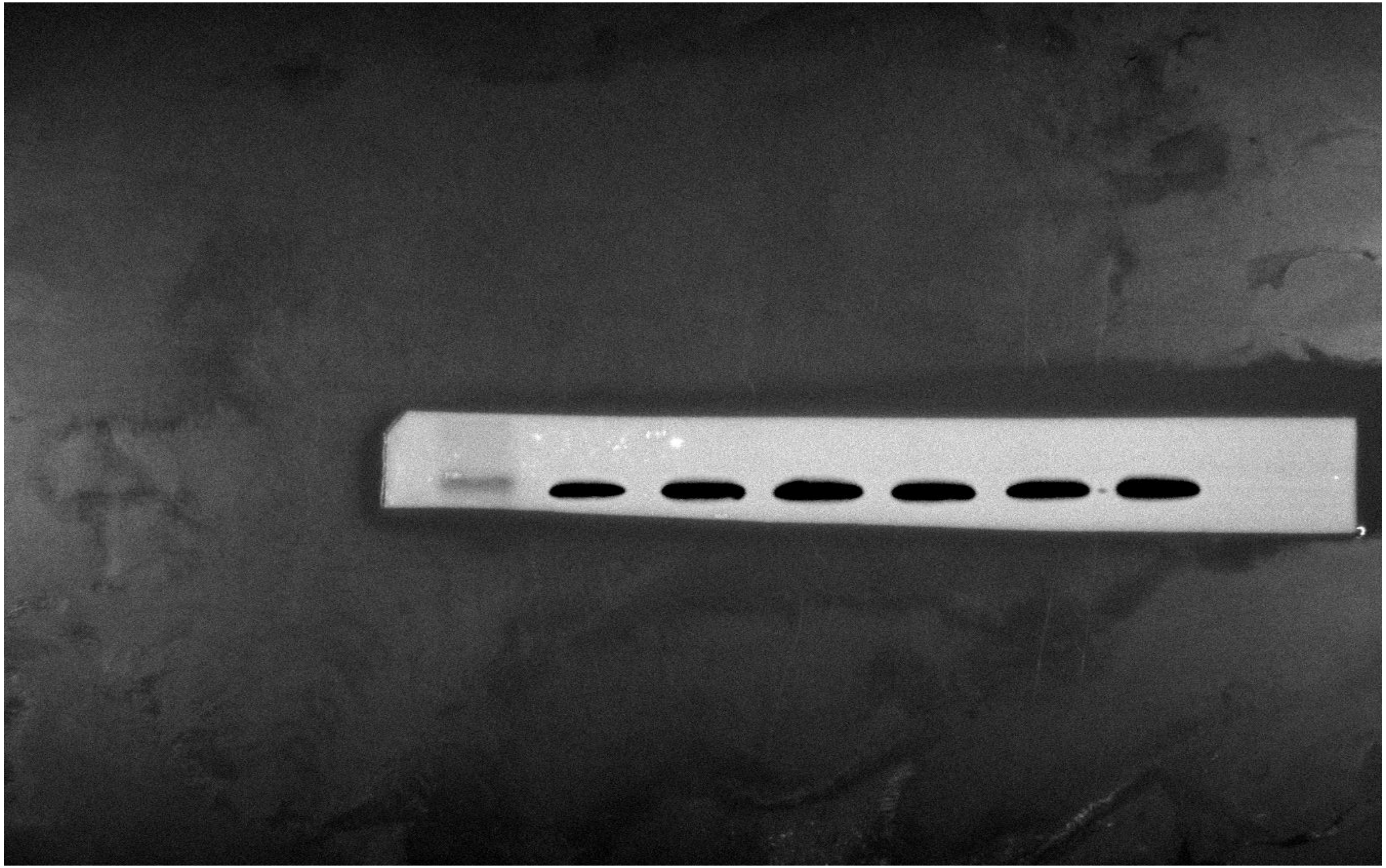

Fig3B **Western blot analysis and quantification of GPX4, SLC7A11, FTH-1, TFR-1 and COX2 in the kidneys of nondiabetic, diabetic and diabetic mice treated with Fer-1.**

COX2

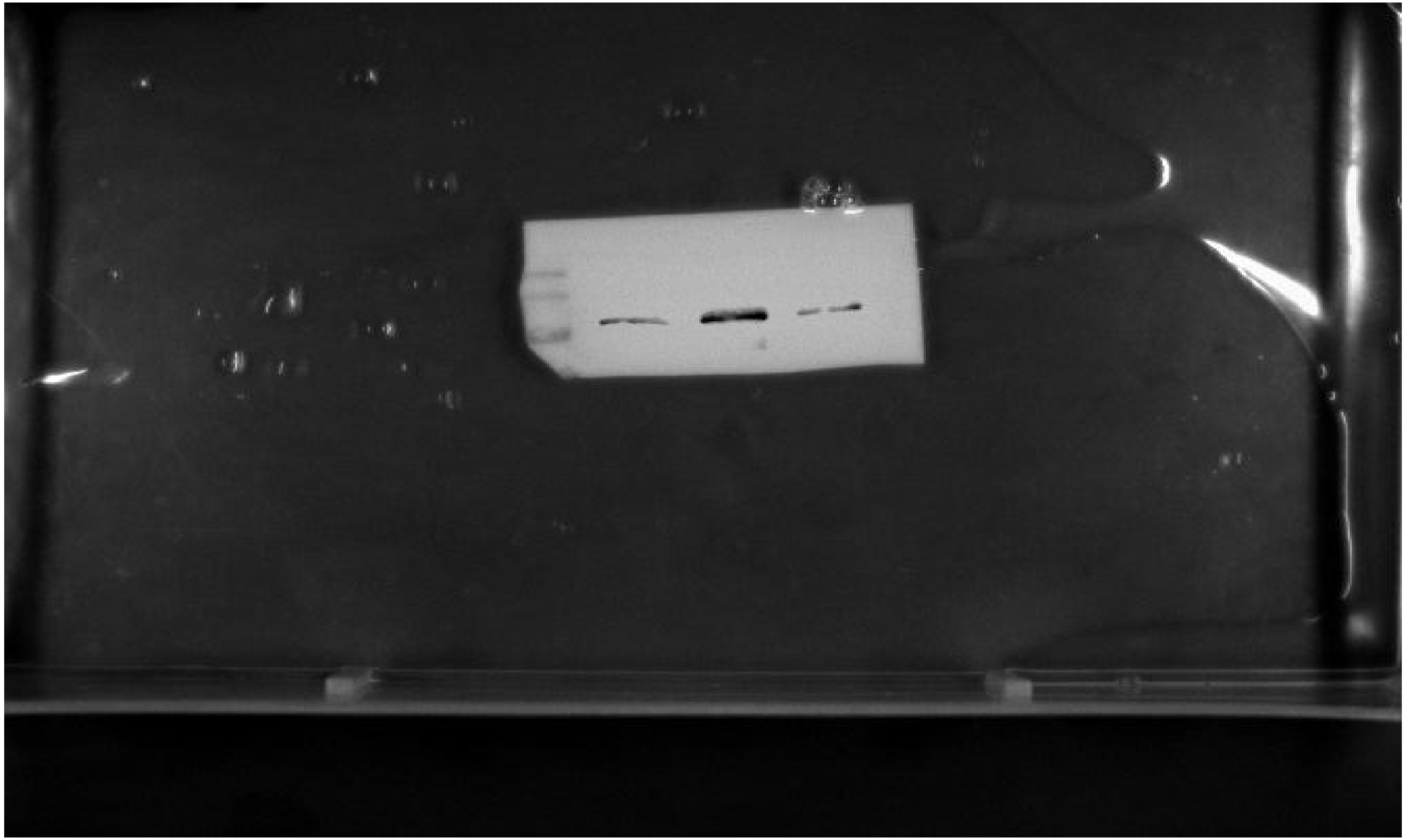

Fig3B

TFR-1

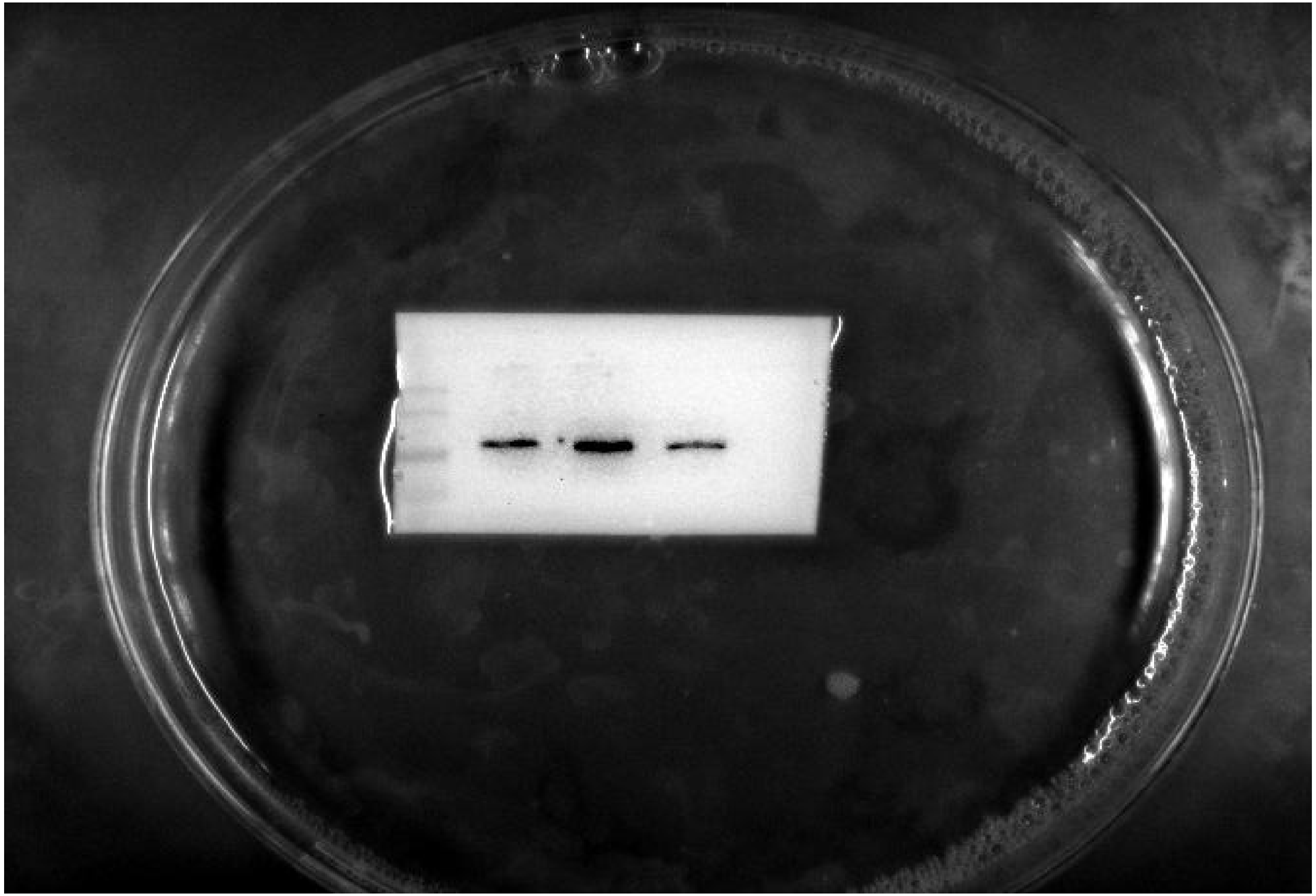

Fig3B

SLC7A11

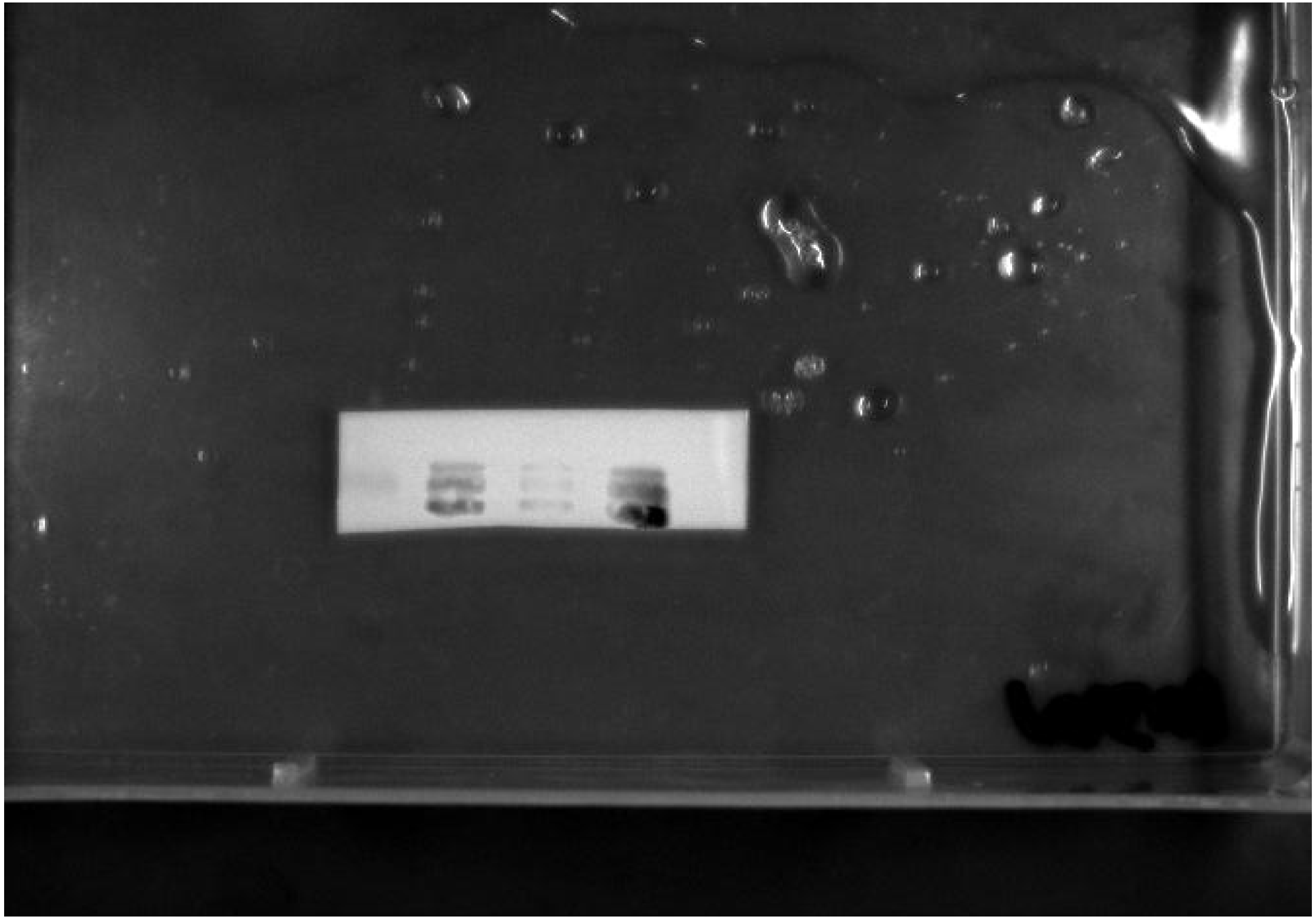

Fig3B

FTH-1

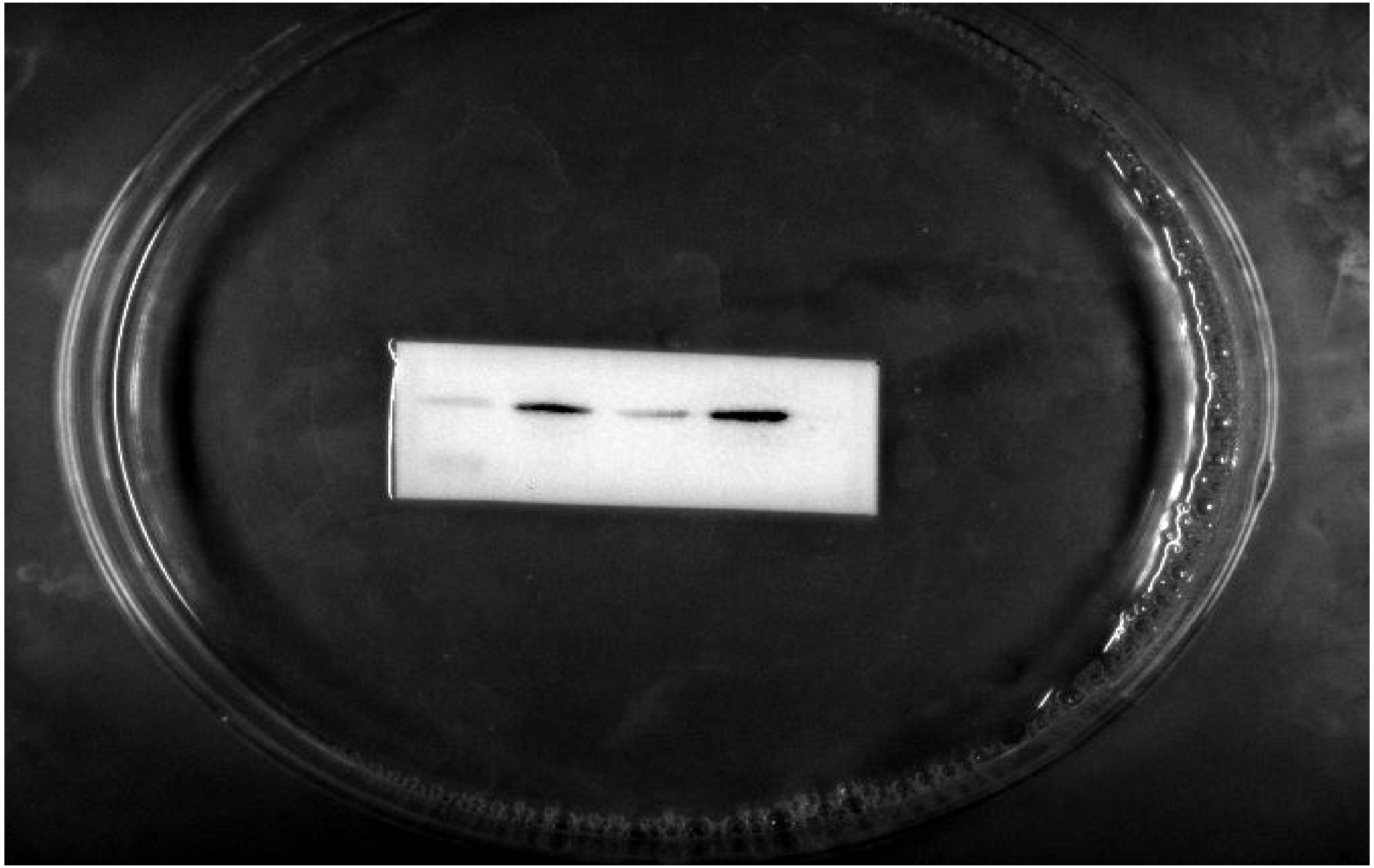

Fig3B

GPX4

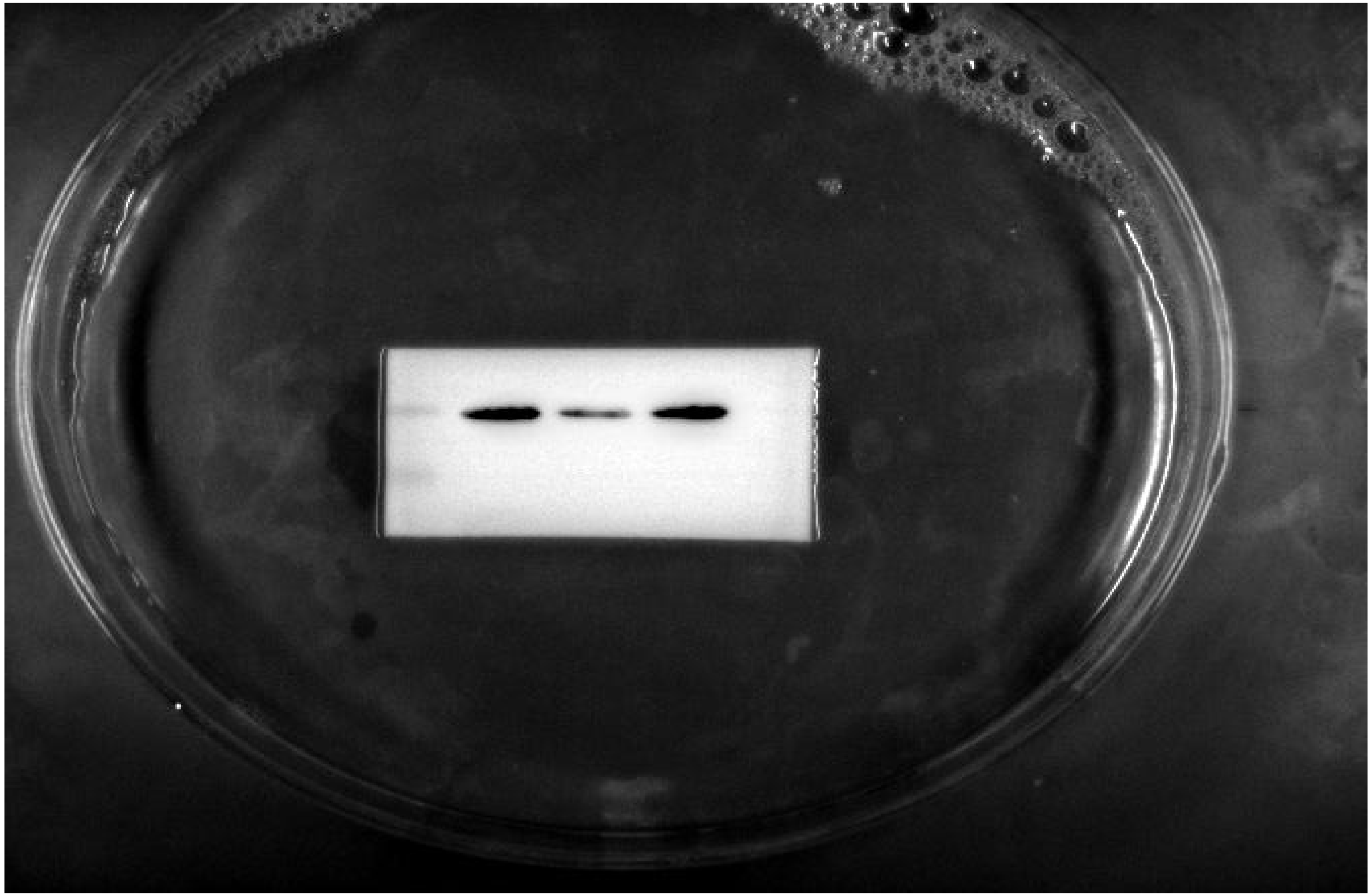

Fig3B

GAPDH

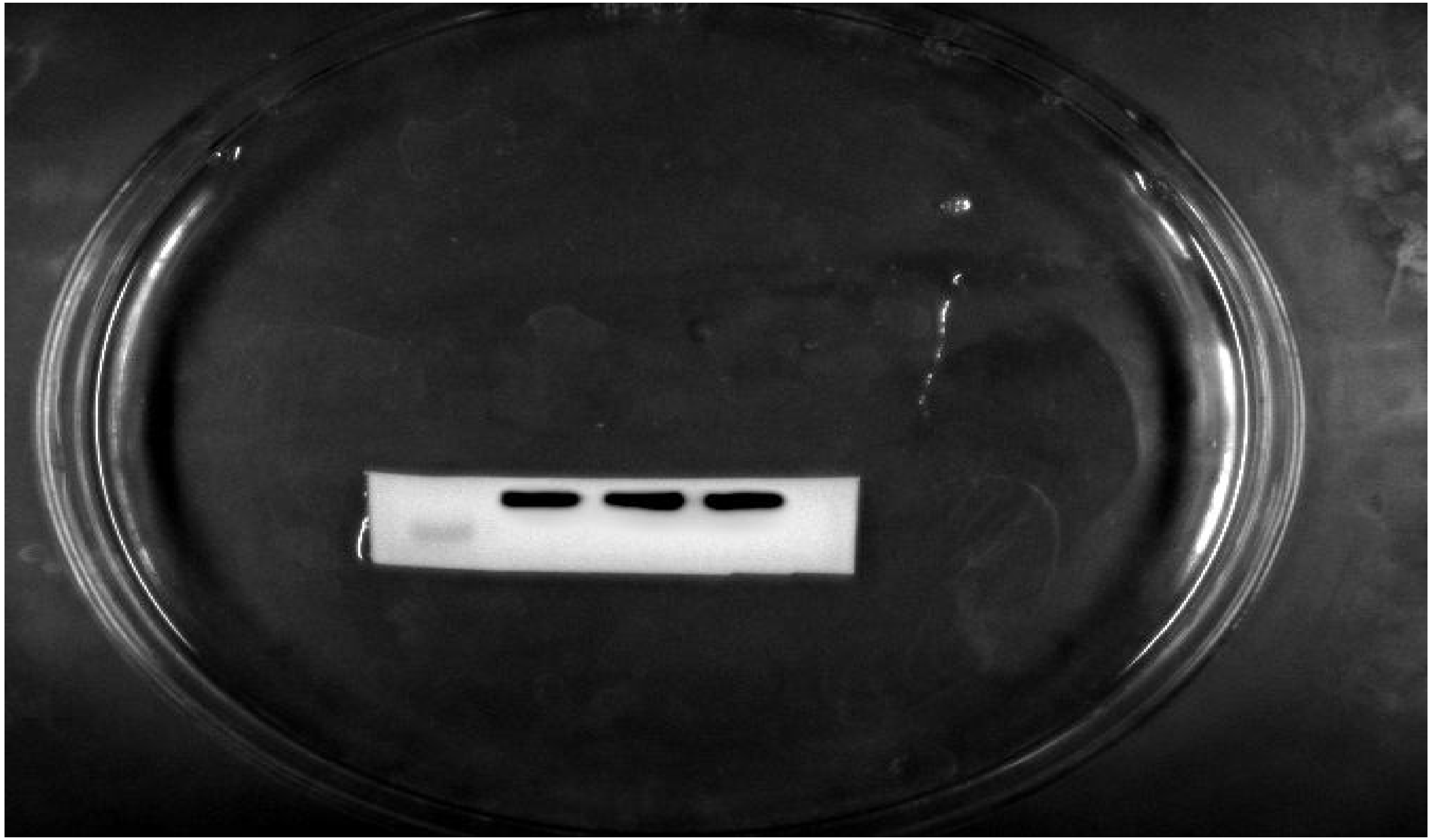

Fig4B **Western blot analysis of GPX4, SLC7A11, FTH-1, TFR-1 and COX2 protein expression in cells in the Ctrl, HG, Fer-1, DMSO and AS groups.**

COX2

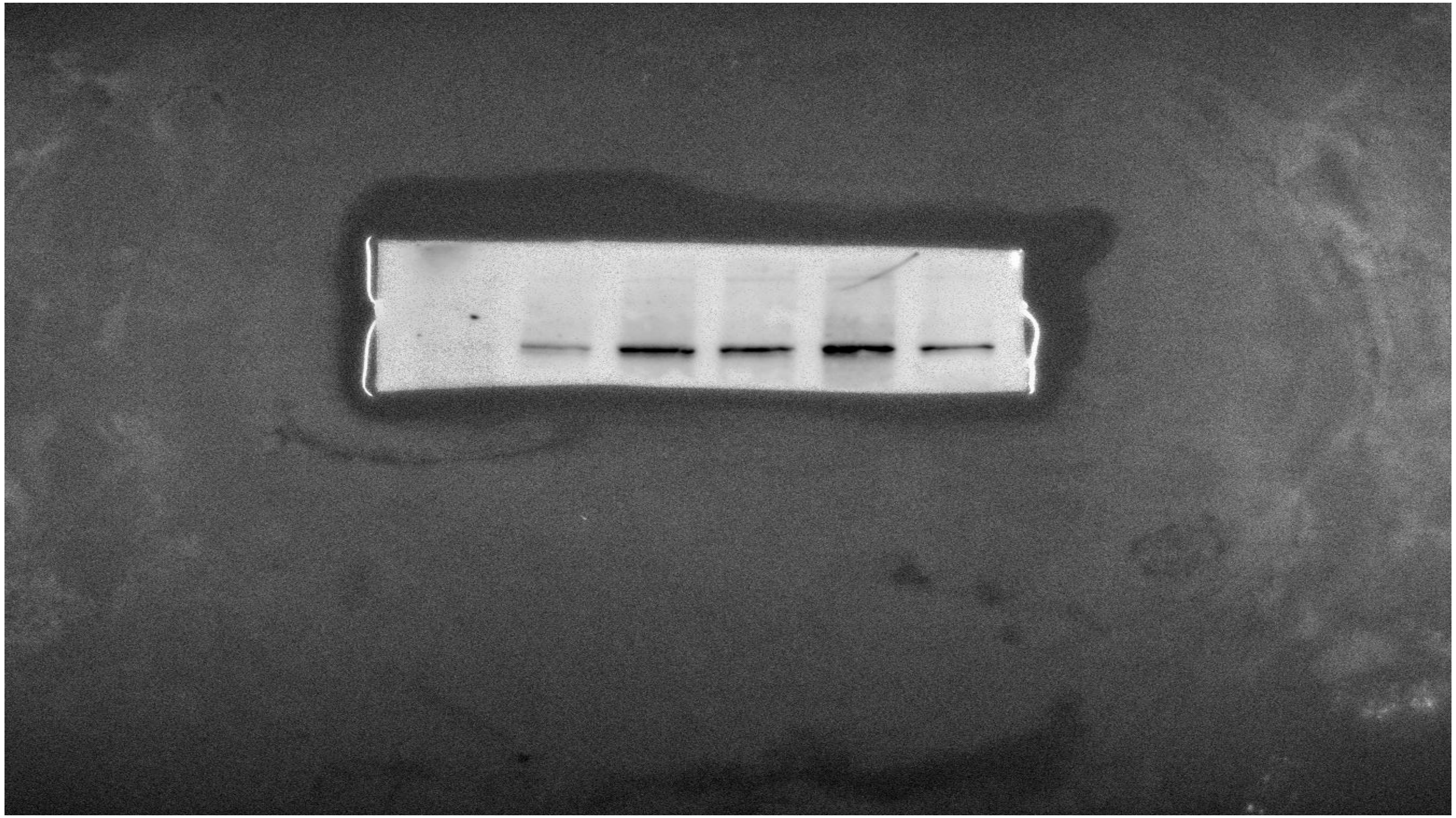

Fig4B

TFR-1

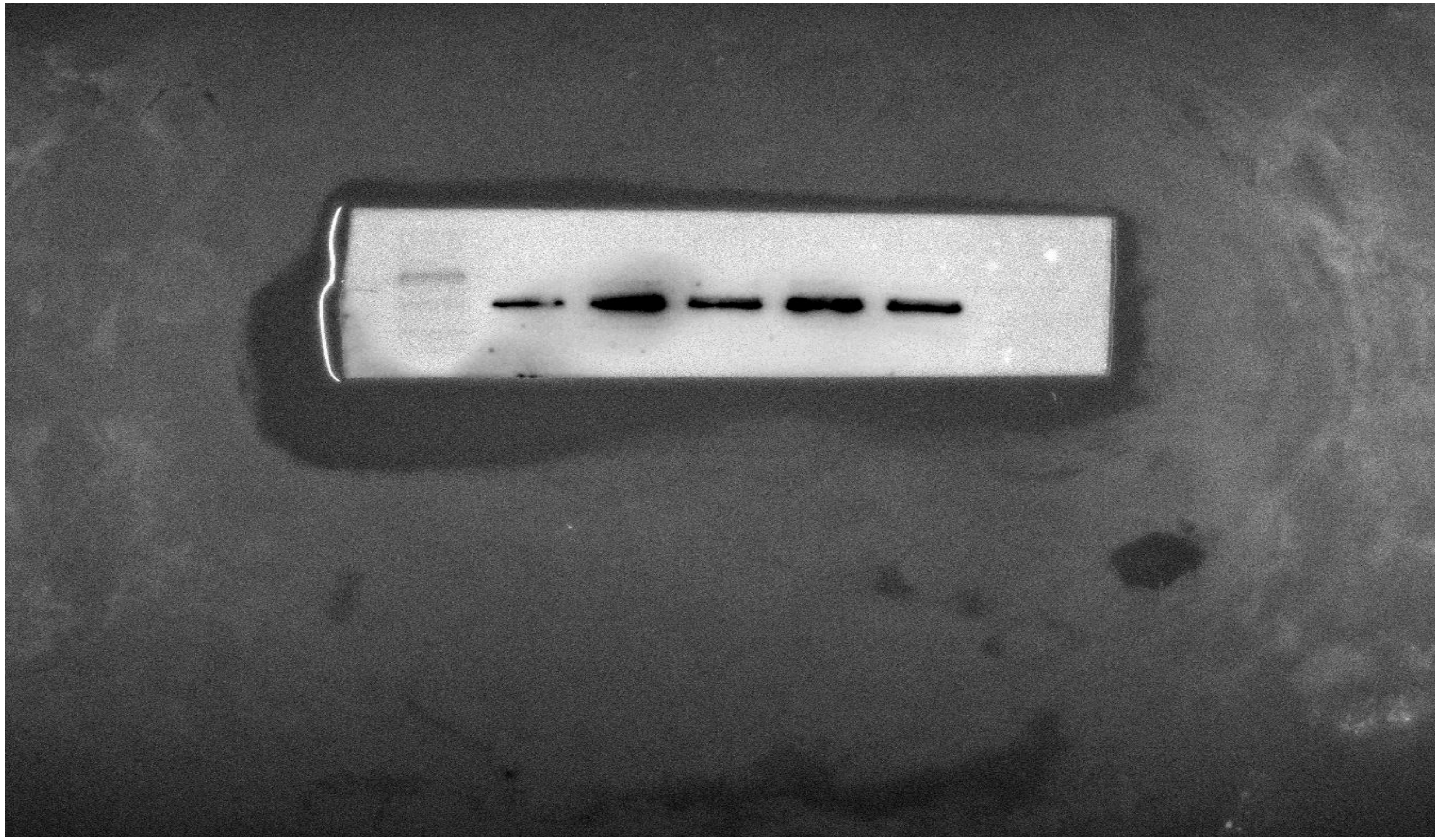

Fig4B

SLC7A11

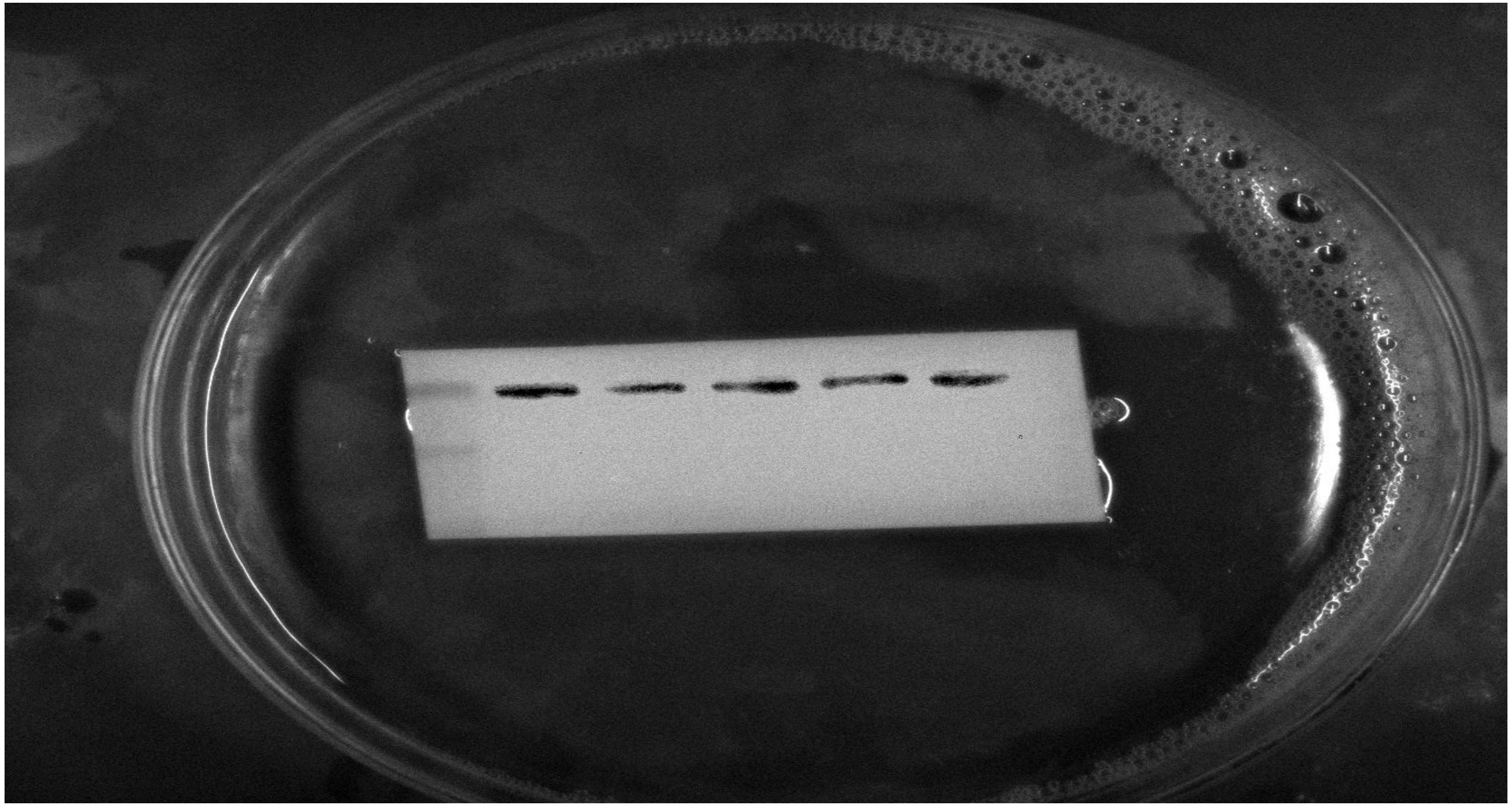

Fig4B

FTH-1

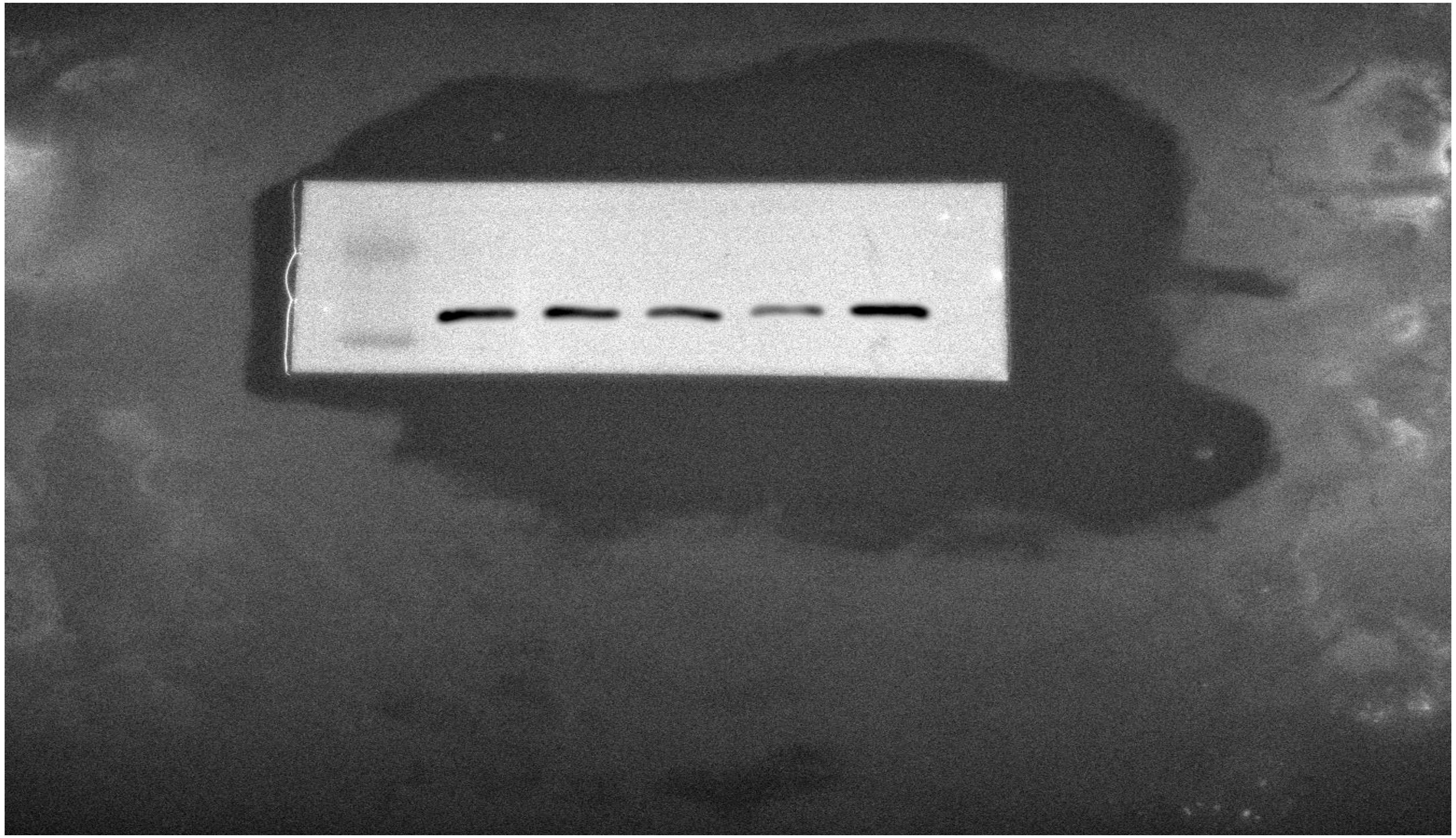

Fig4B

GPX4

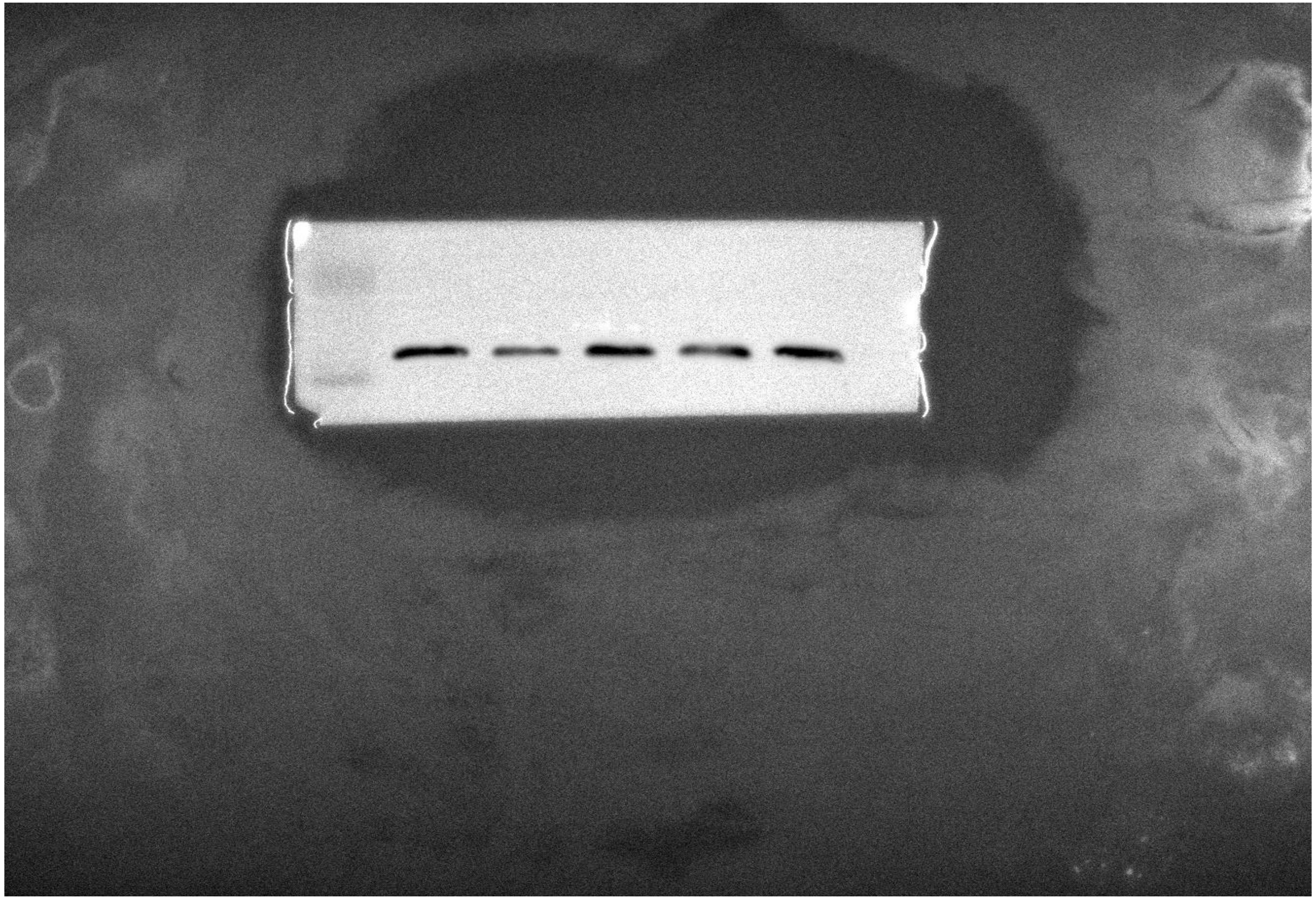

Fig4B

GAPDH

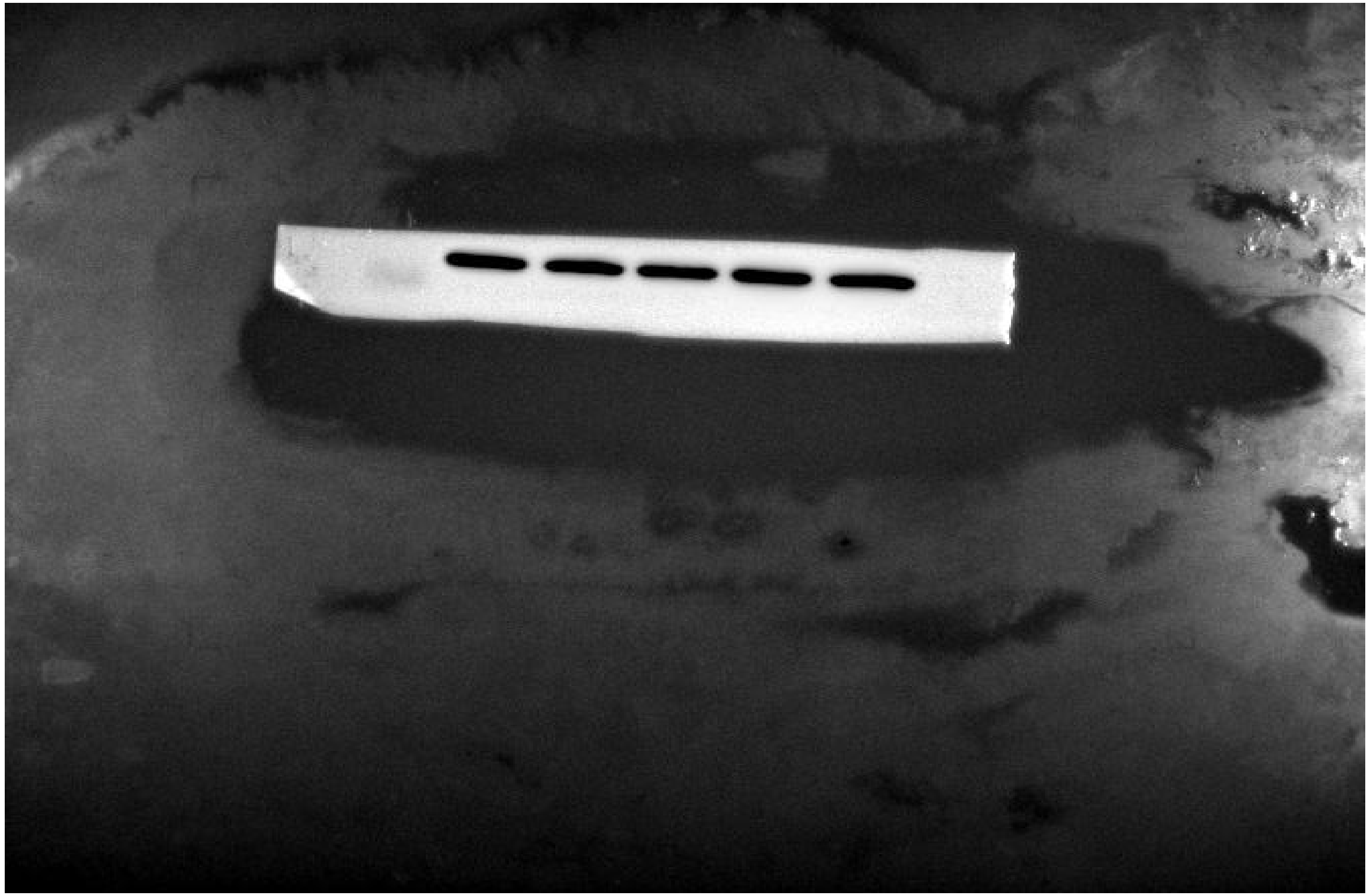

Fig 5B **Western blotting showed the protein expression of COX2, GPX4, SLC7A11, FTH-1 and TFR-1 in the cells of each group.**

COX2

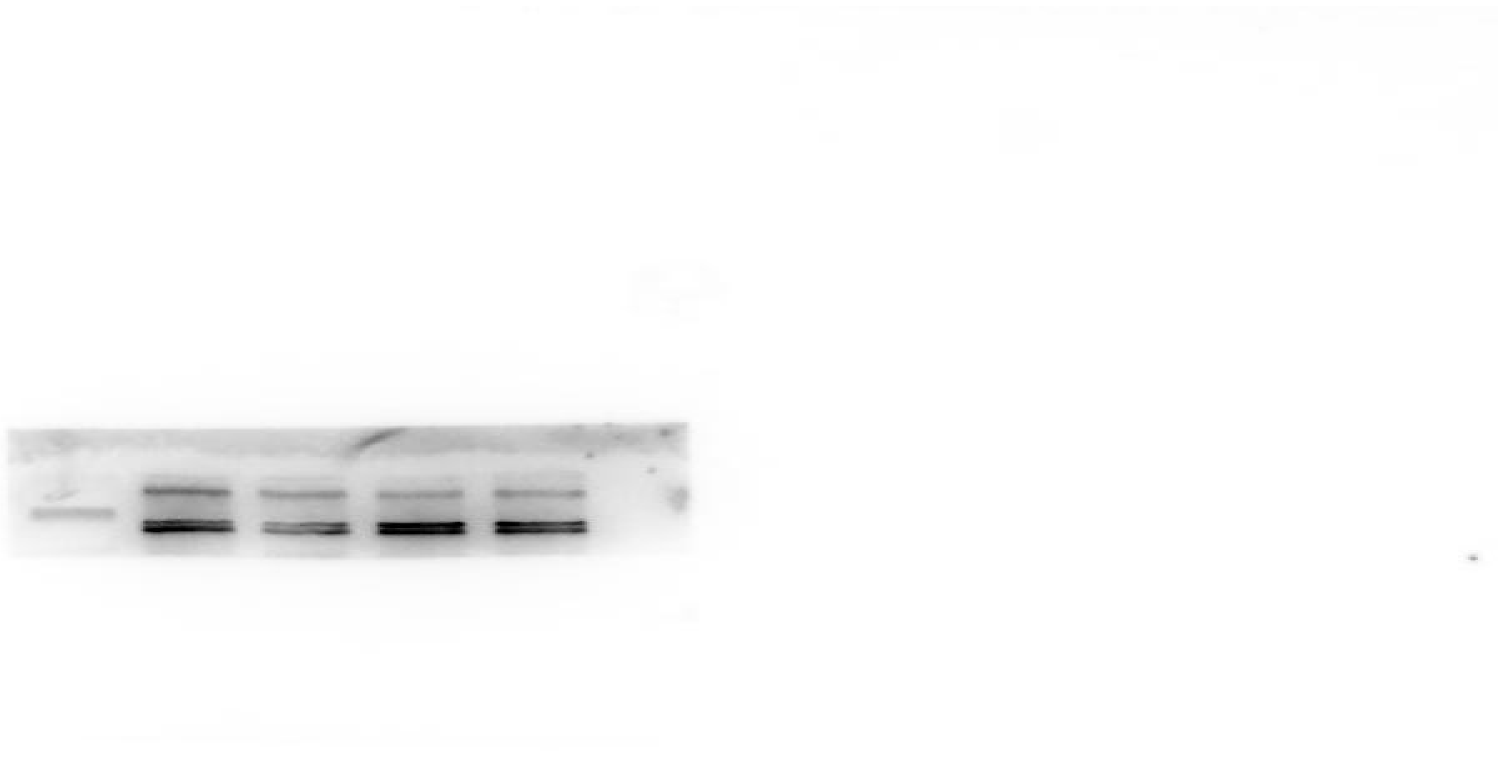

Fig 5B

TFR-1

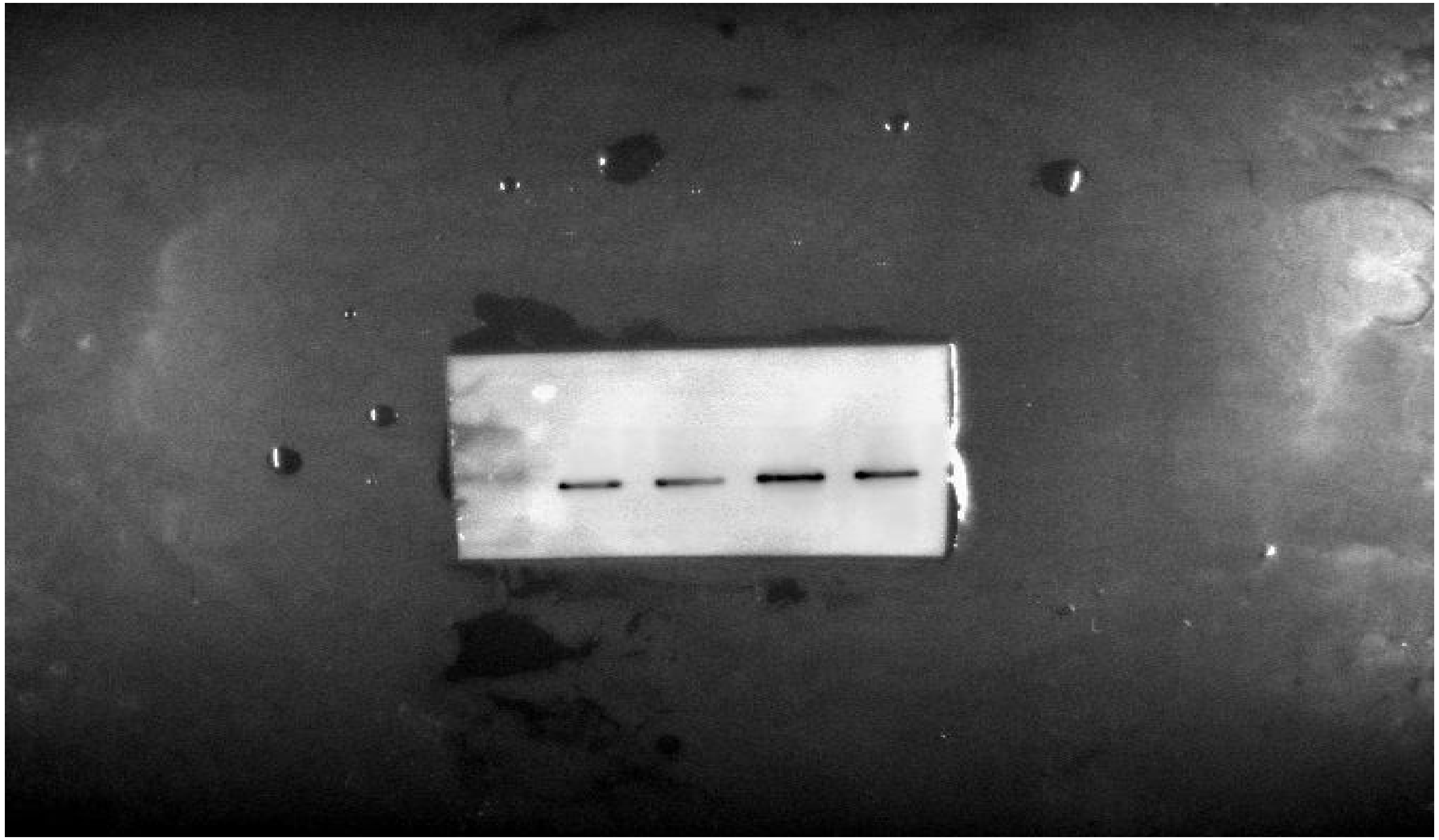

Fig 5B

SLC7A11

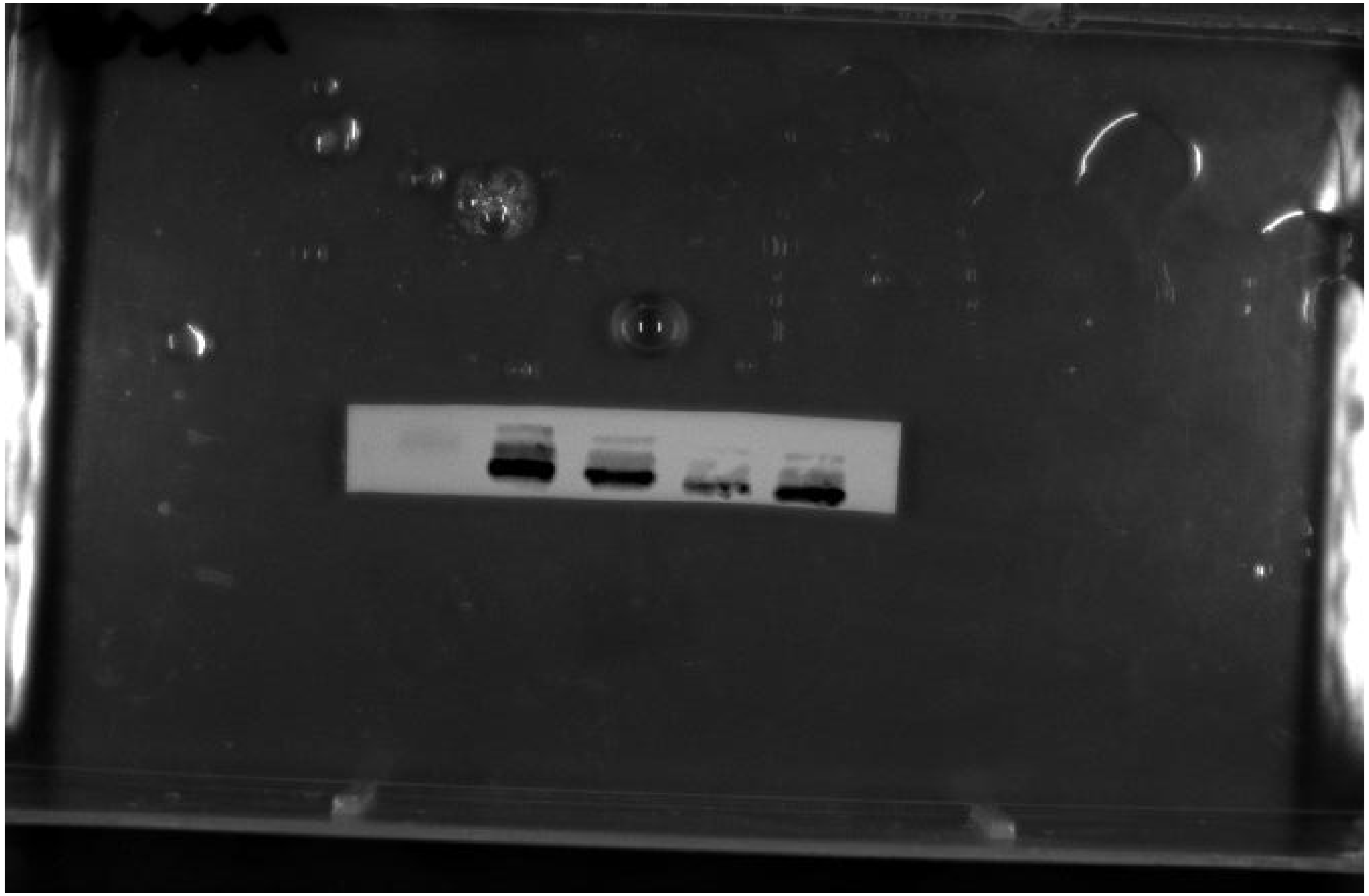

Fig 5B

FTH-1

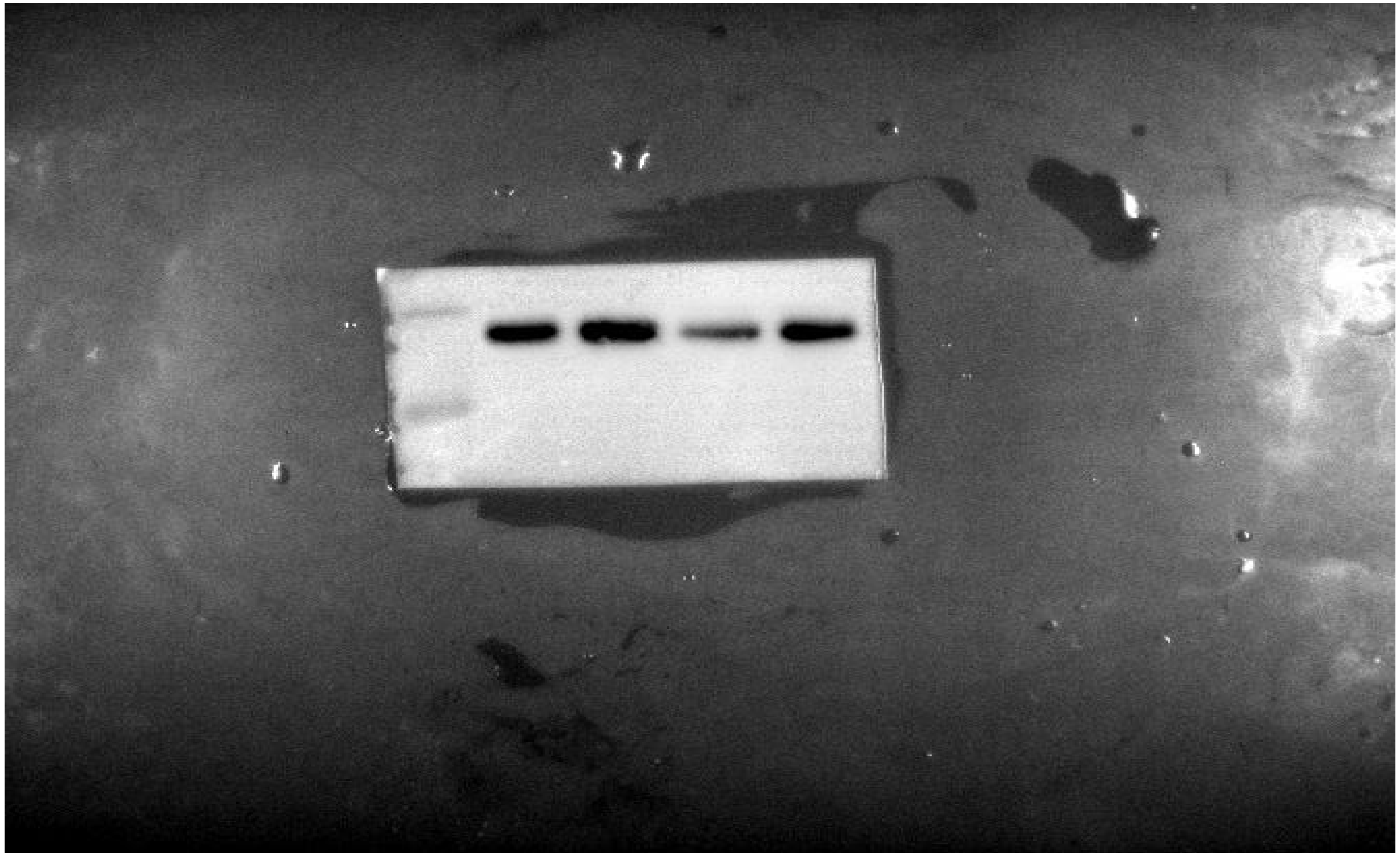

Fig 5B

GPX4

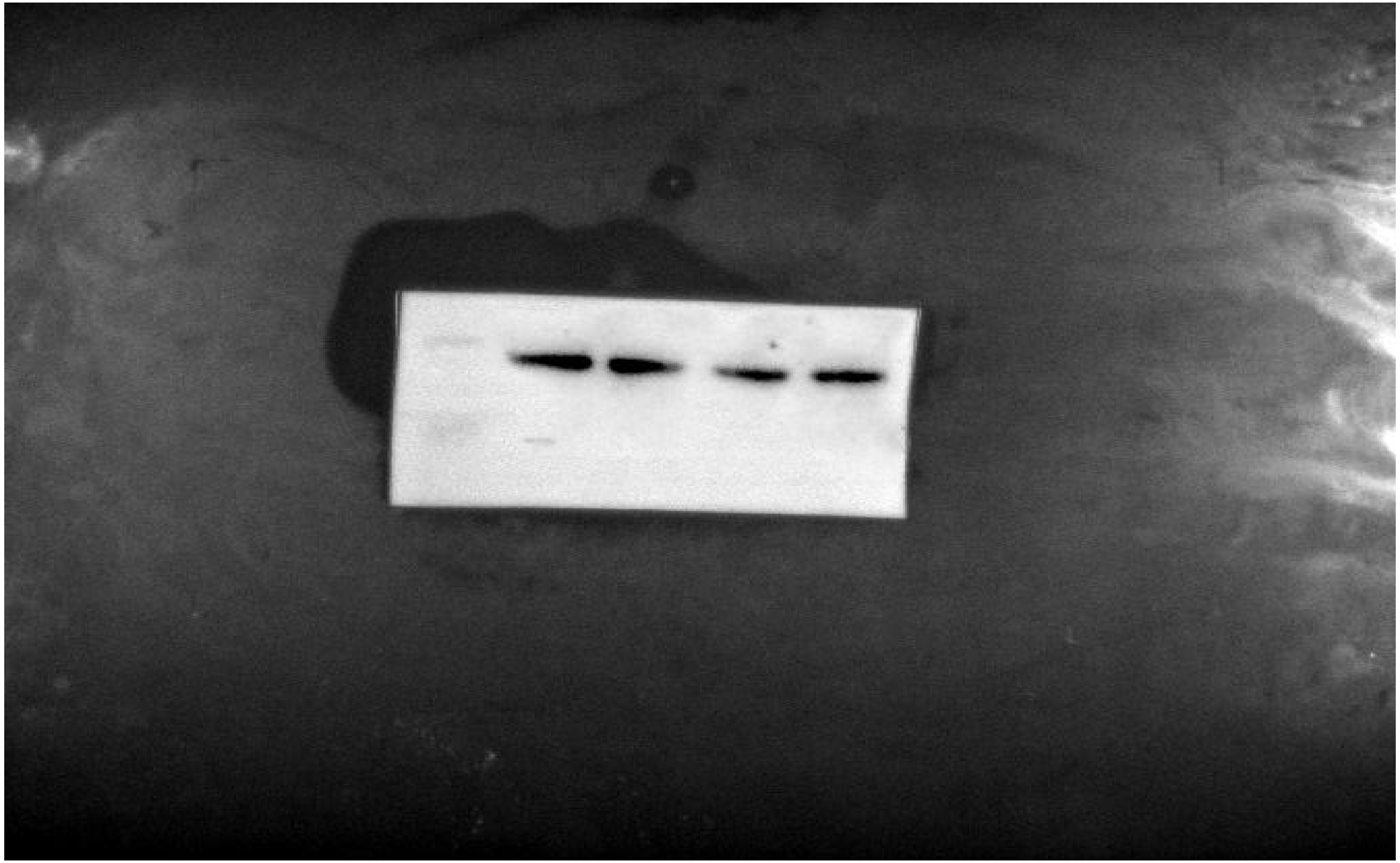

Fig 5B

GAPDH

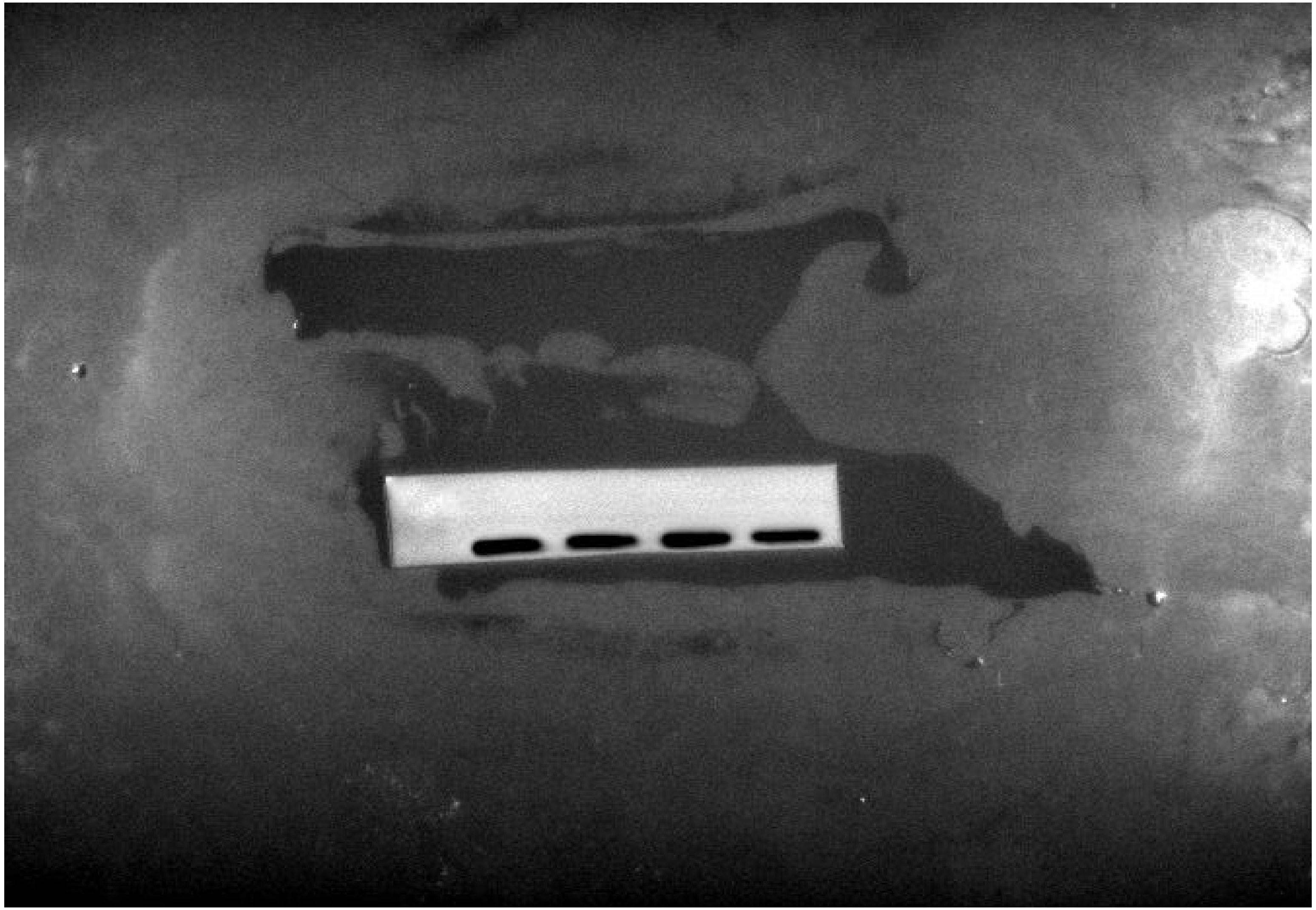

Fig6D

Western blot analysis of SLC7A11, GPX4, FTH-1, TFR-1 and COX2 protein expression of cells in Ctrl, DMSO, RSL-3 100 nm, RSL-3 100 nm+Fer-1 400 nm， RSL-3 100 nm+AS400 µm and AS 400 µm groups.

COX2

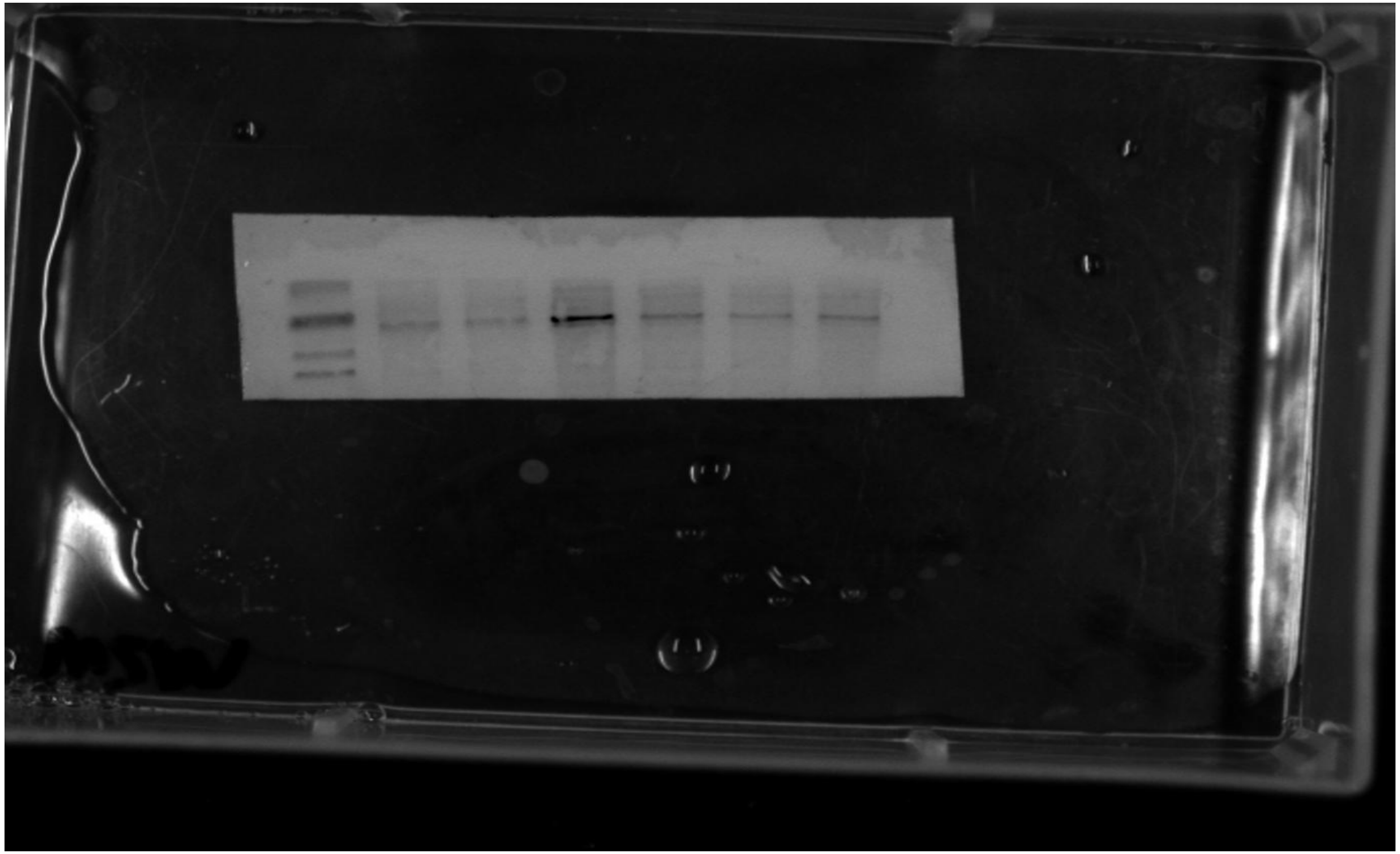

Fig6D

TFR-1

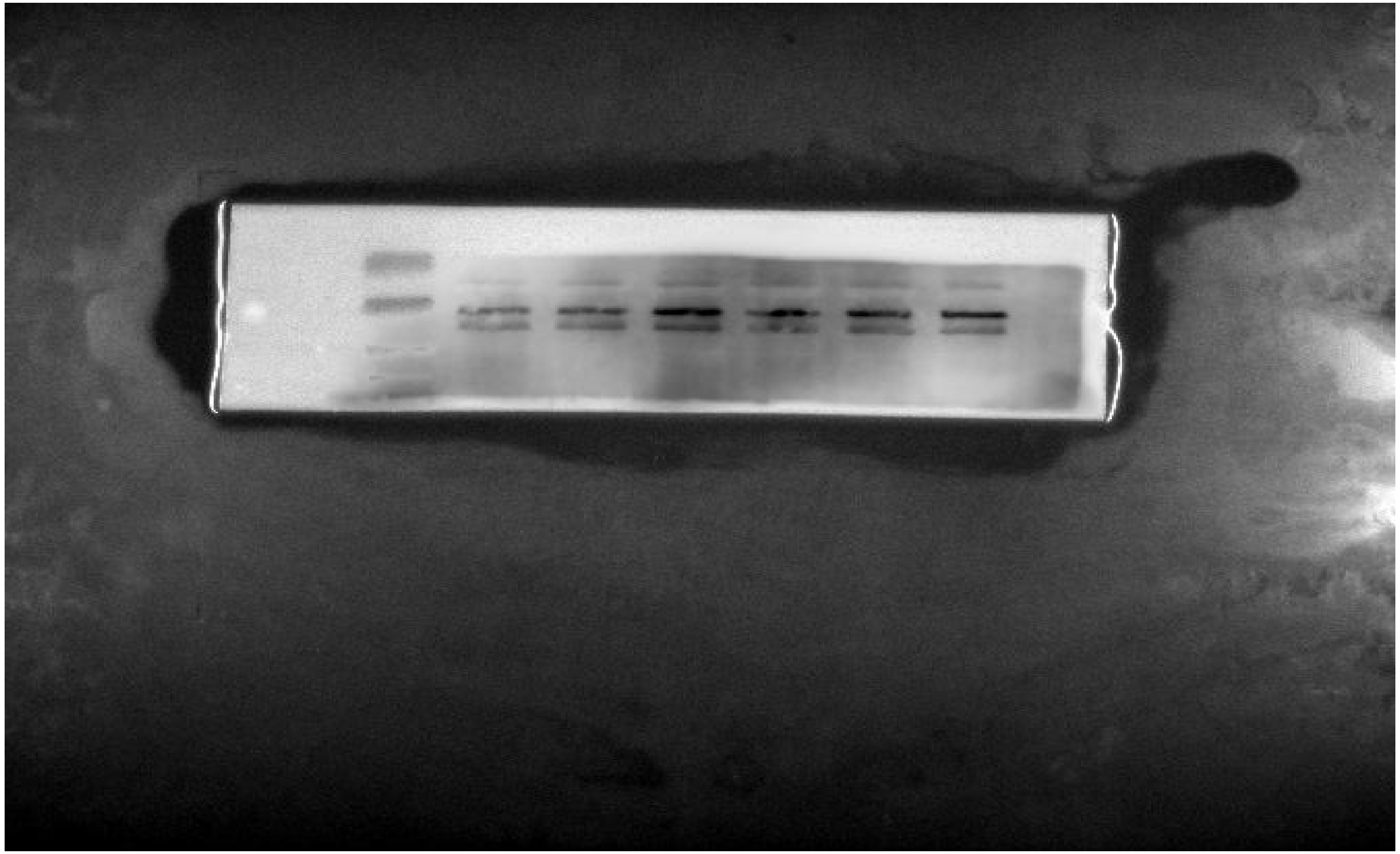

Fig6D

SLC7A11

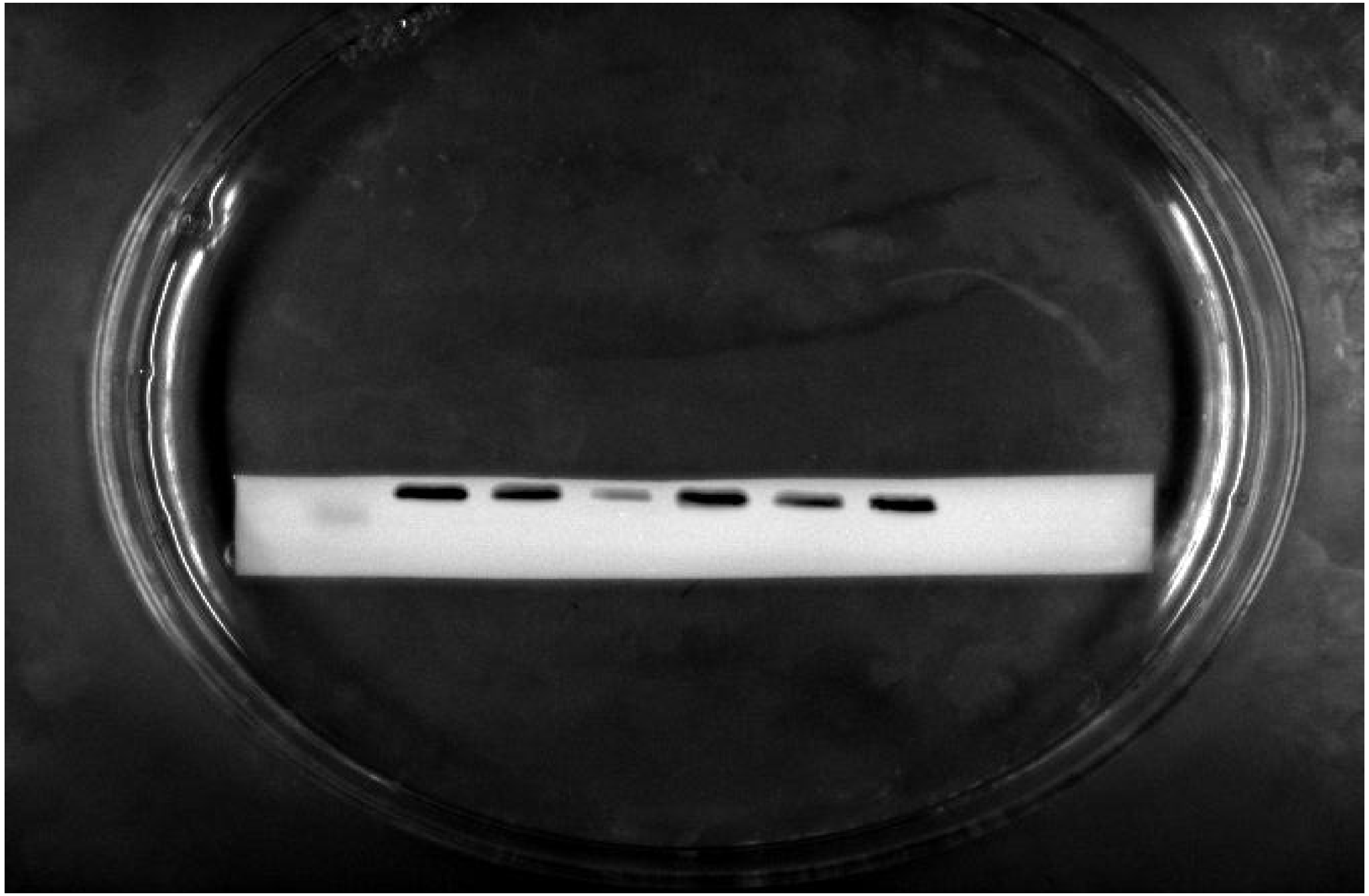

Fig6D

FTH-1

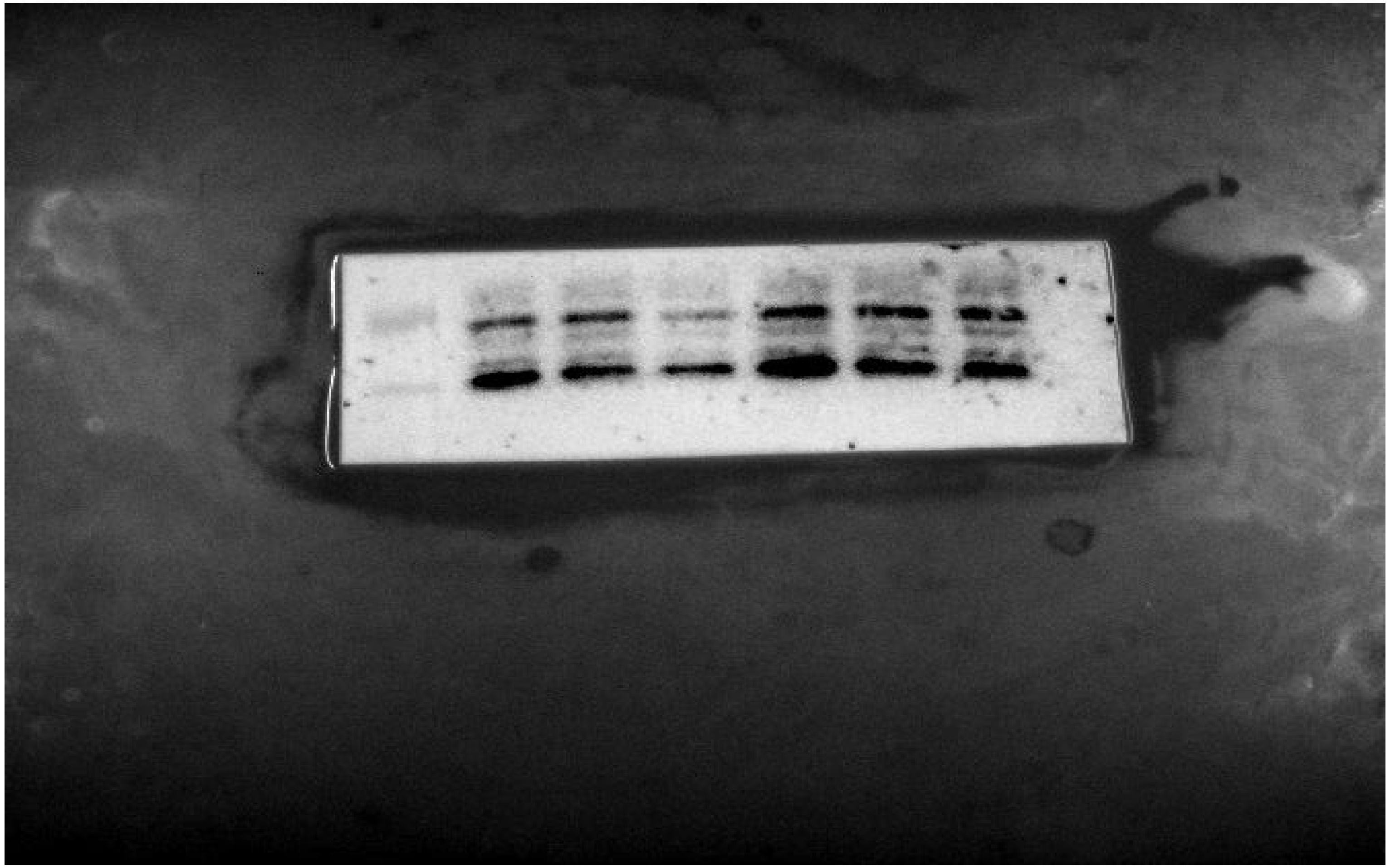

Fig6D

GPX4

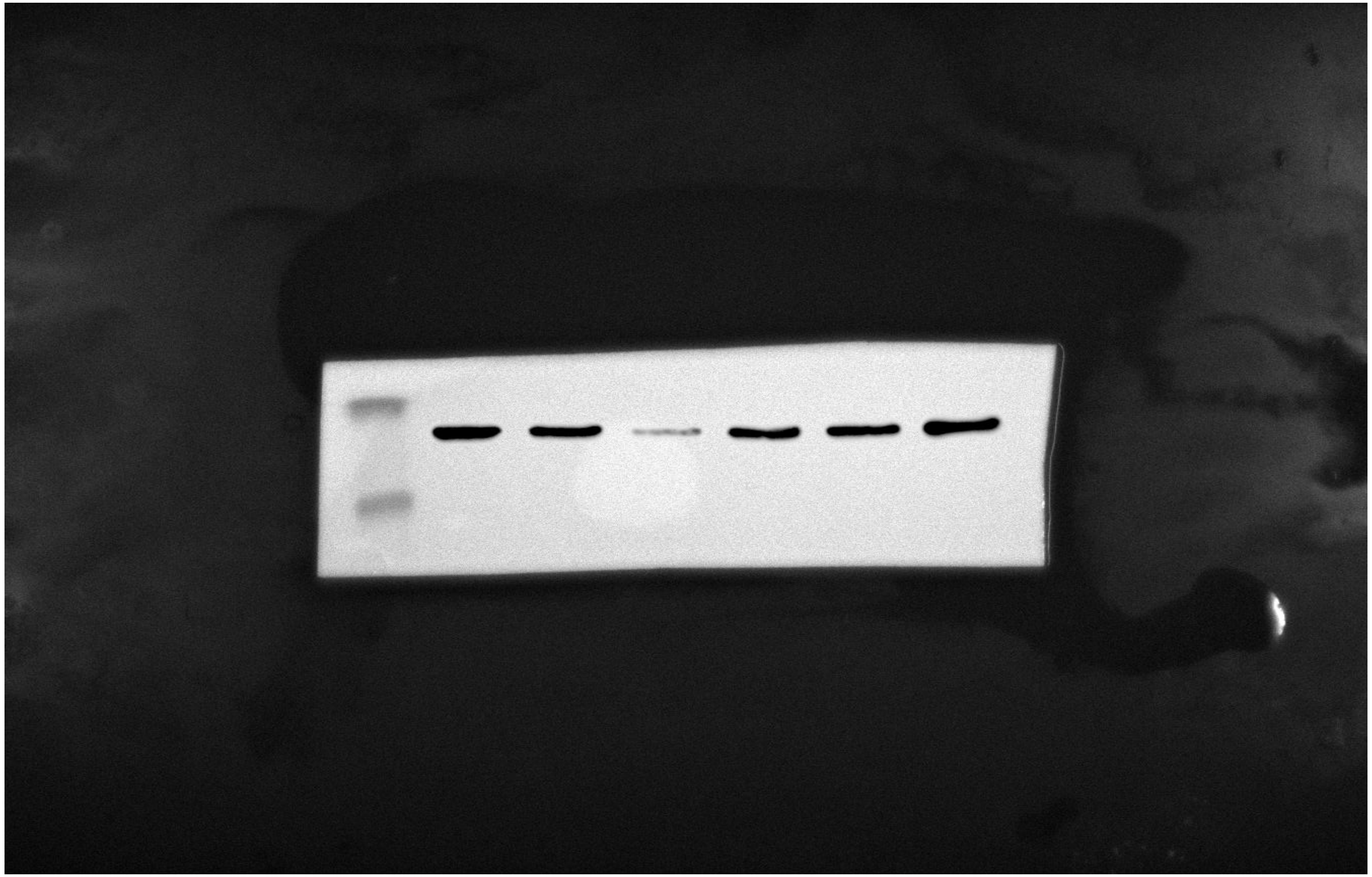

Fig6D

GAPDH

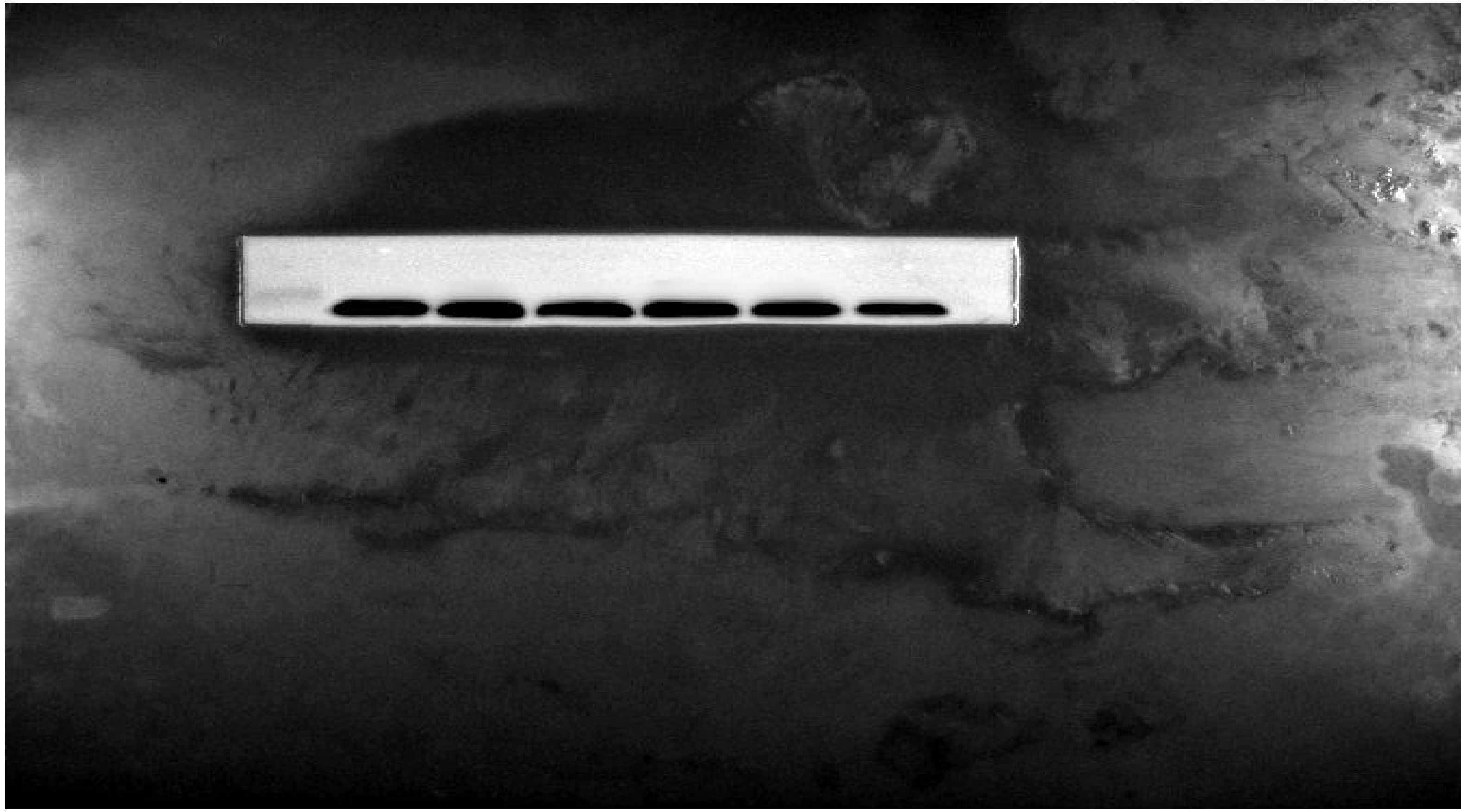

Fig7C **Western blotting results of COX2, GPX4, SLC7A11, FTH-1 and TFR-1 in cells in each group.**

COX2

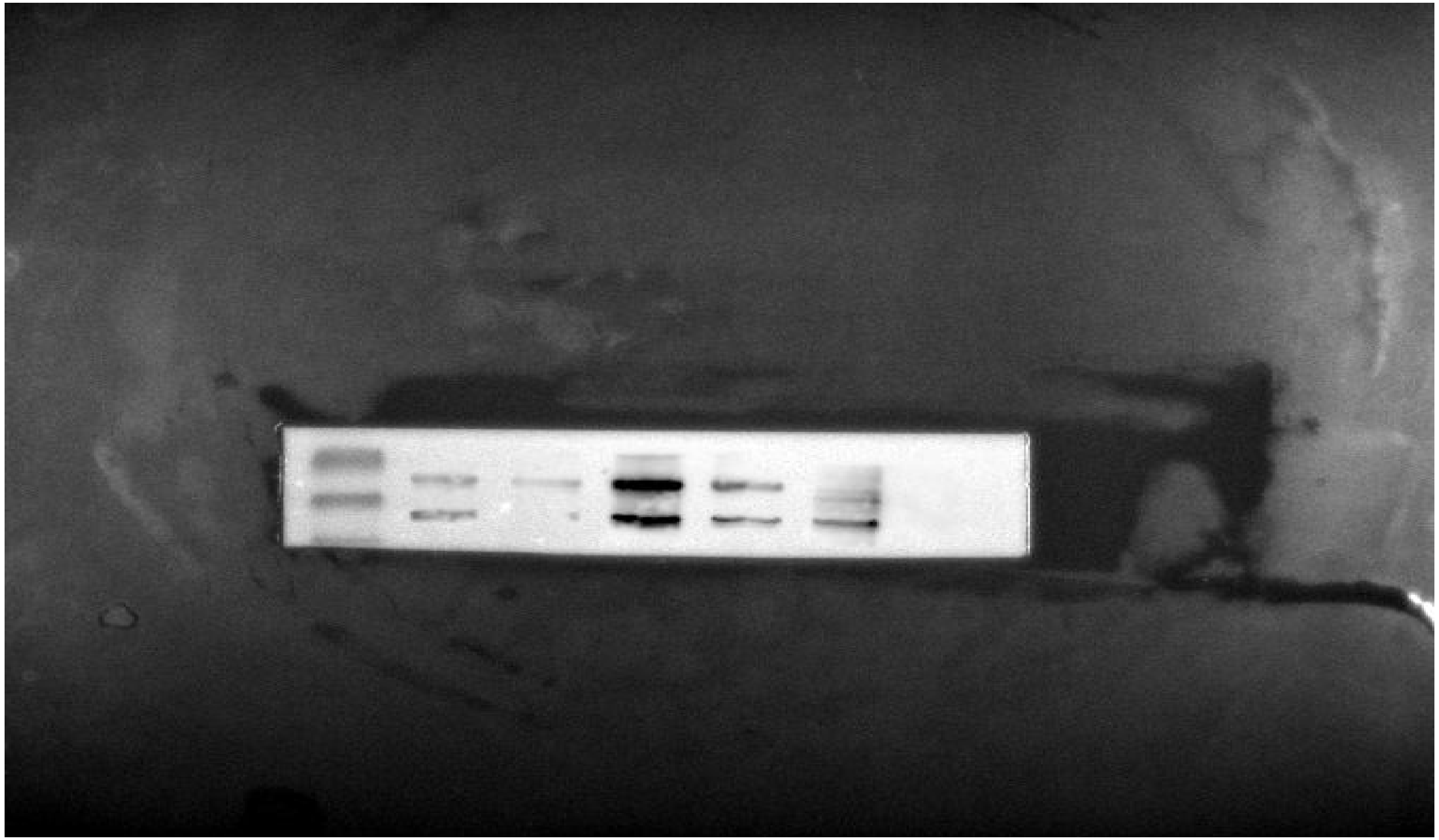

Fig7C

TFR-1

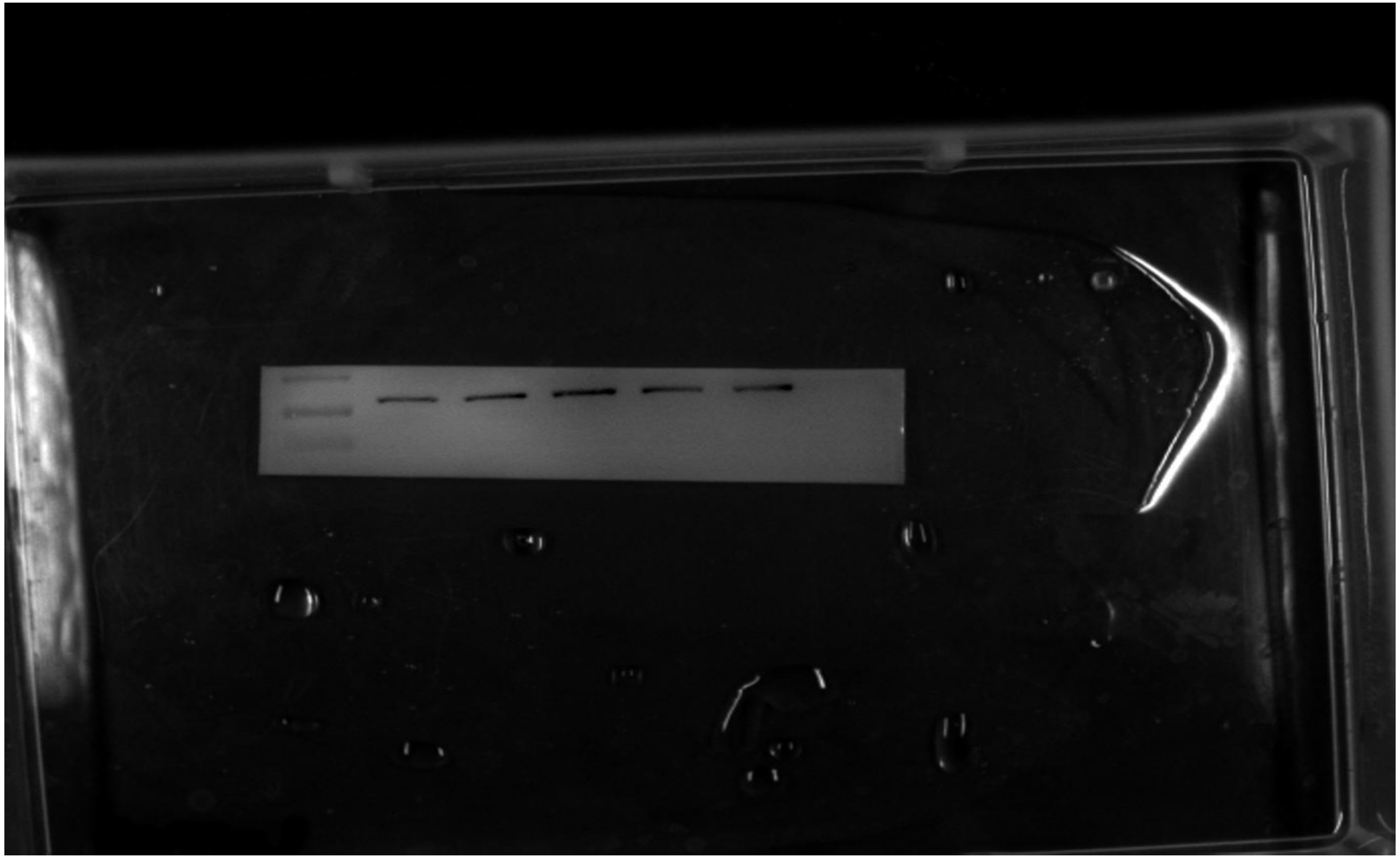

Fig7C

SLC7A11

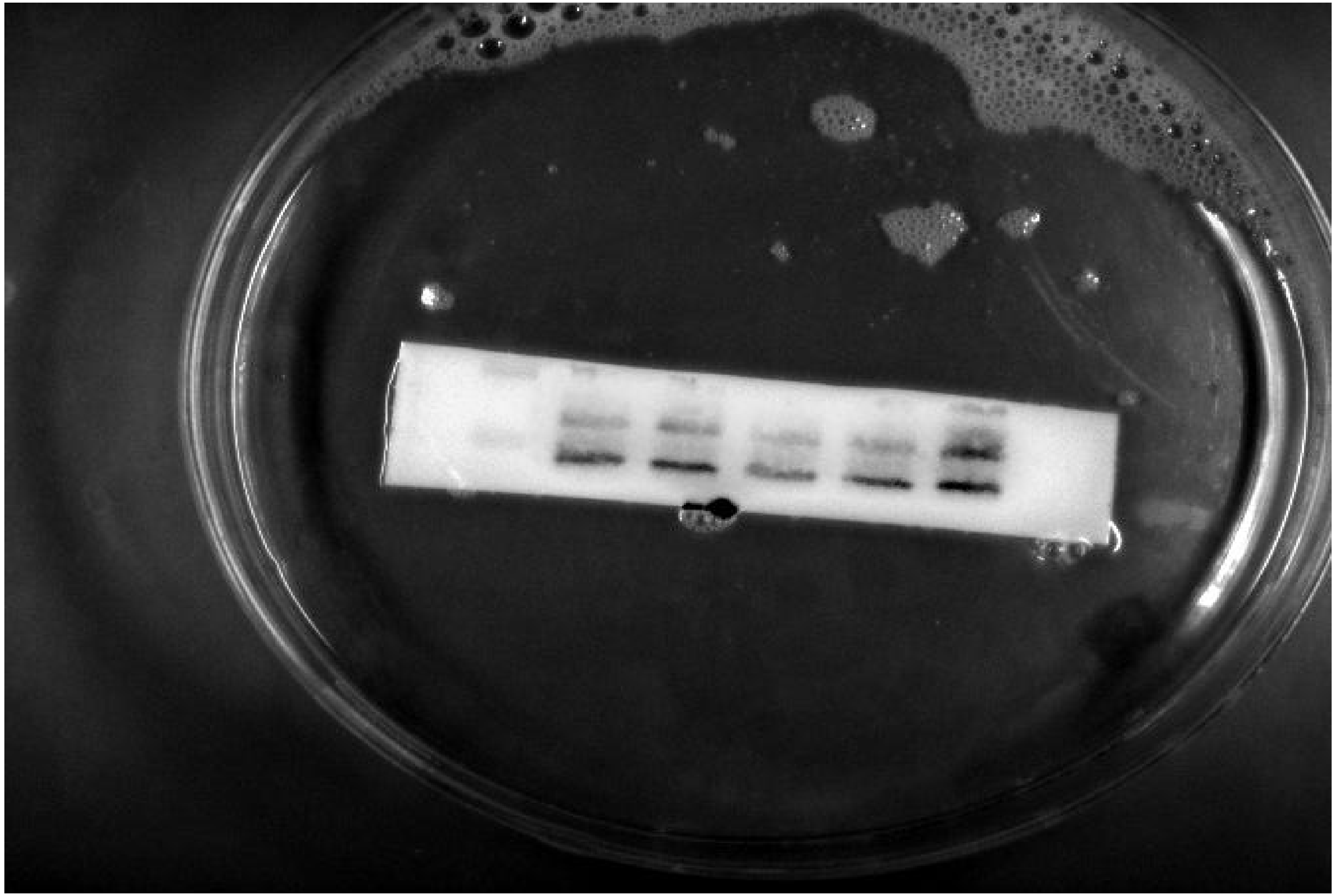

Fig7C

FTH-1

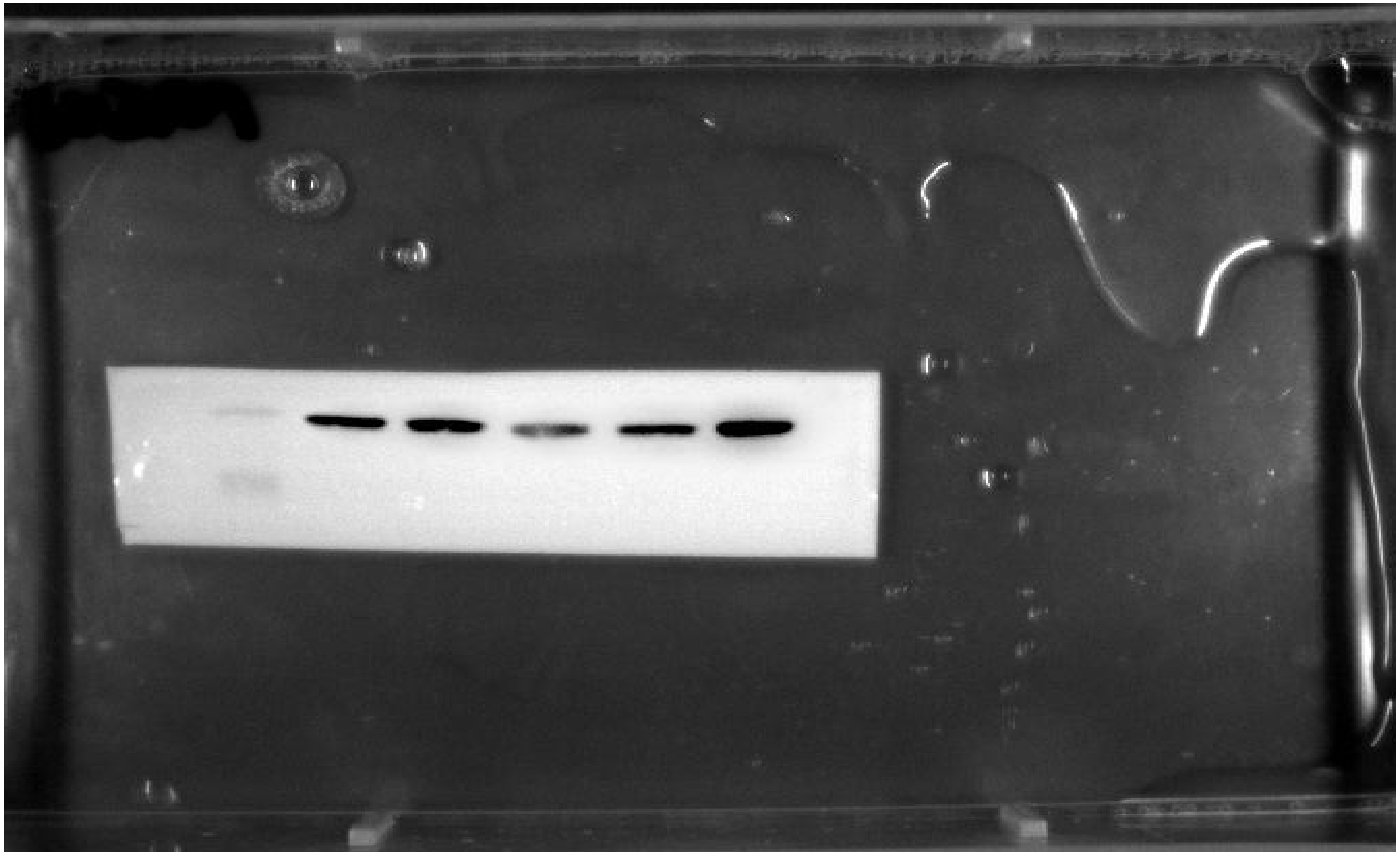

Fig7C

GPX4

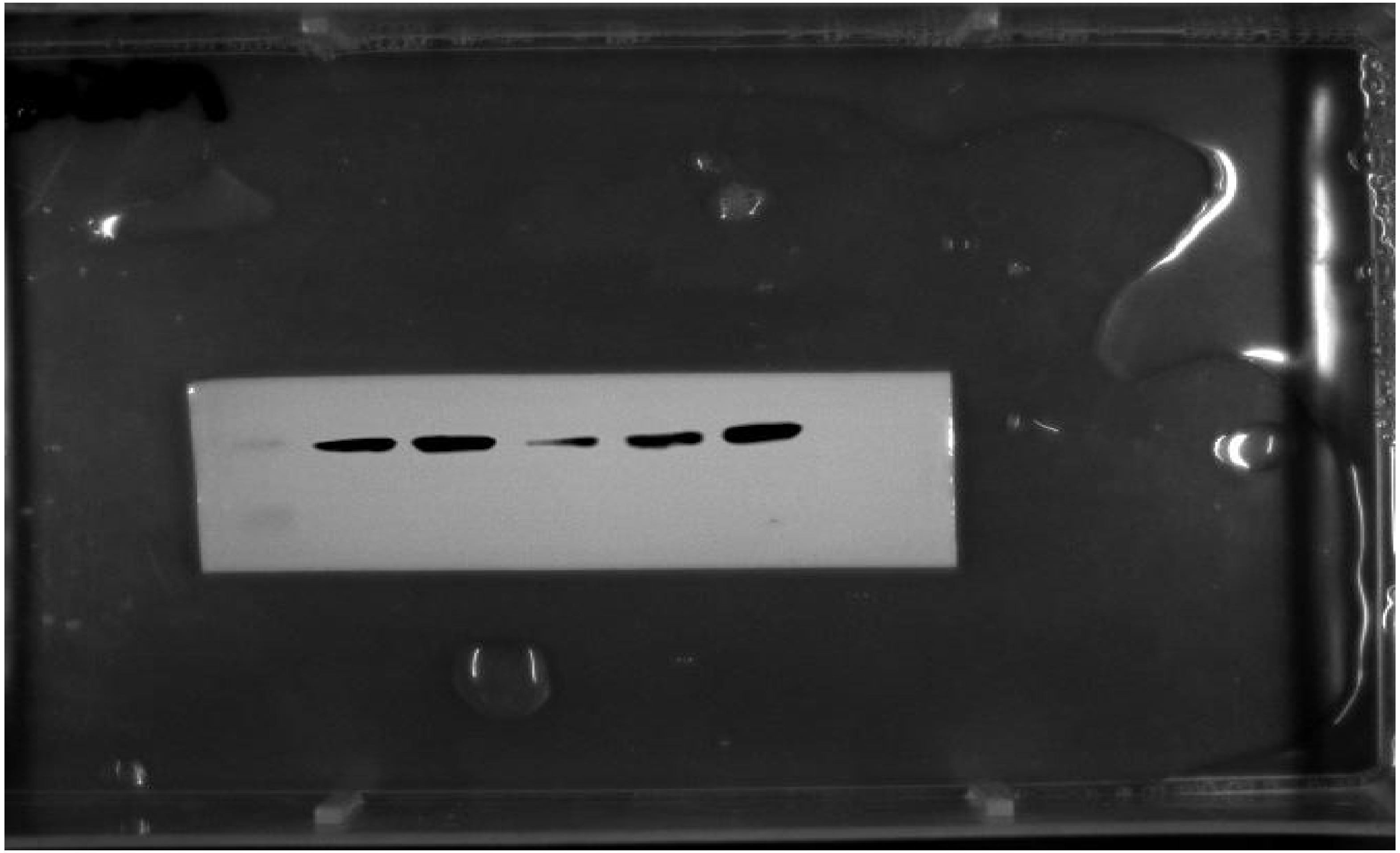

Fig7C

GAPDH

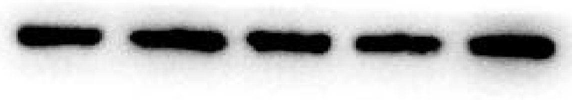

Fig9B **Western blot analysis of GPX4, SLC7A11, FTH-1, TFR-1 and COX2 in the kidneys of nondiabetic, diabetic control and diabetic mice treated with AS.**

COX2

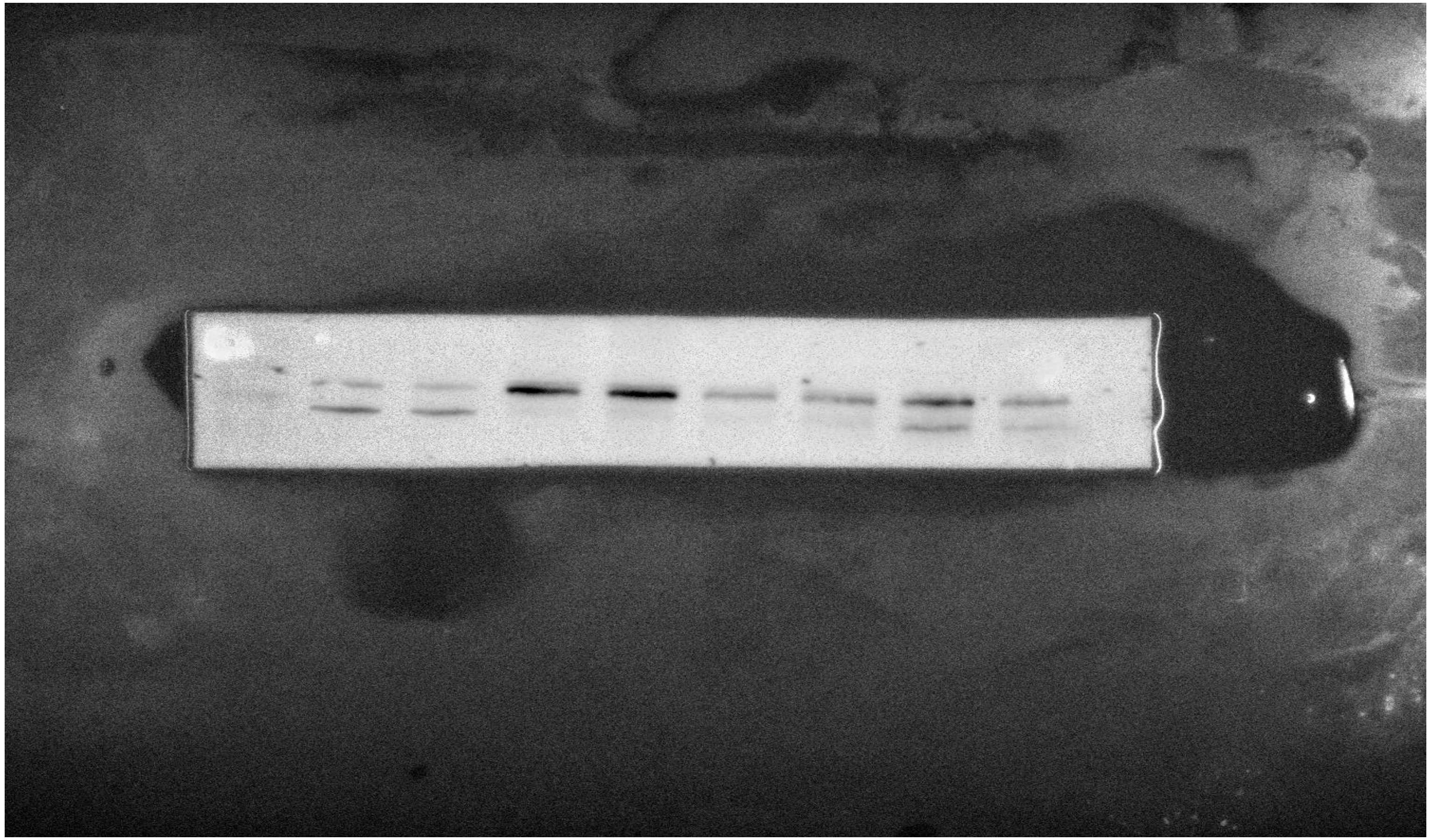

Fig9B

TFR-1

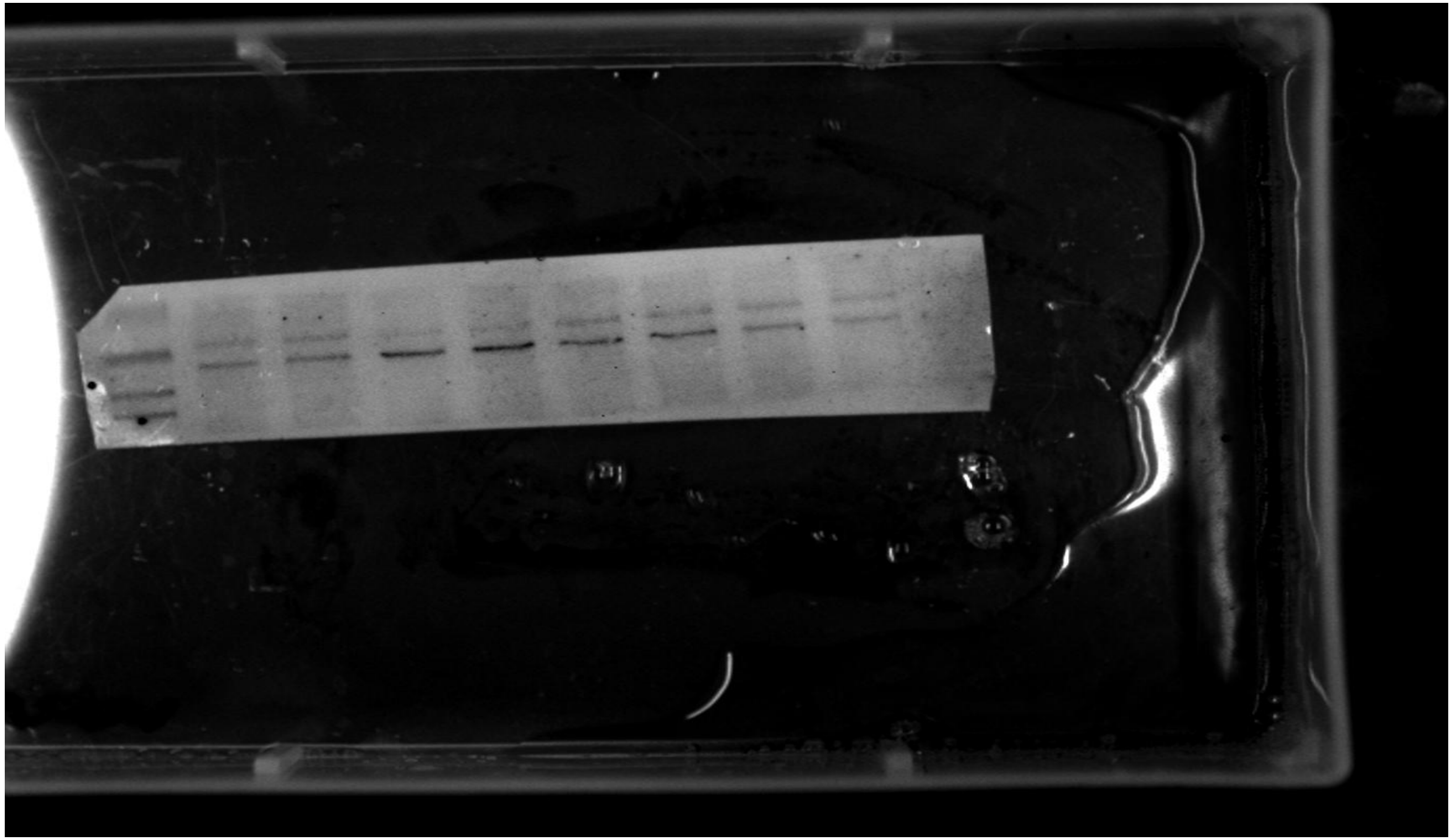

Fig9B

SLC7A11

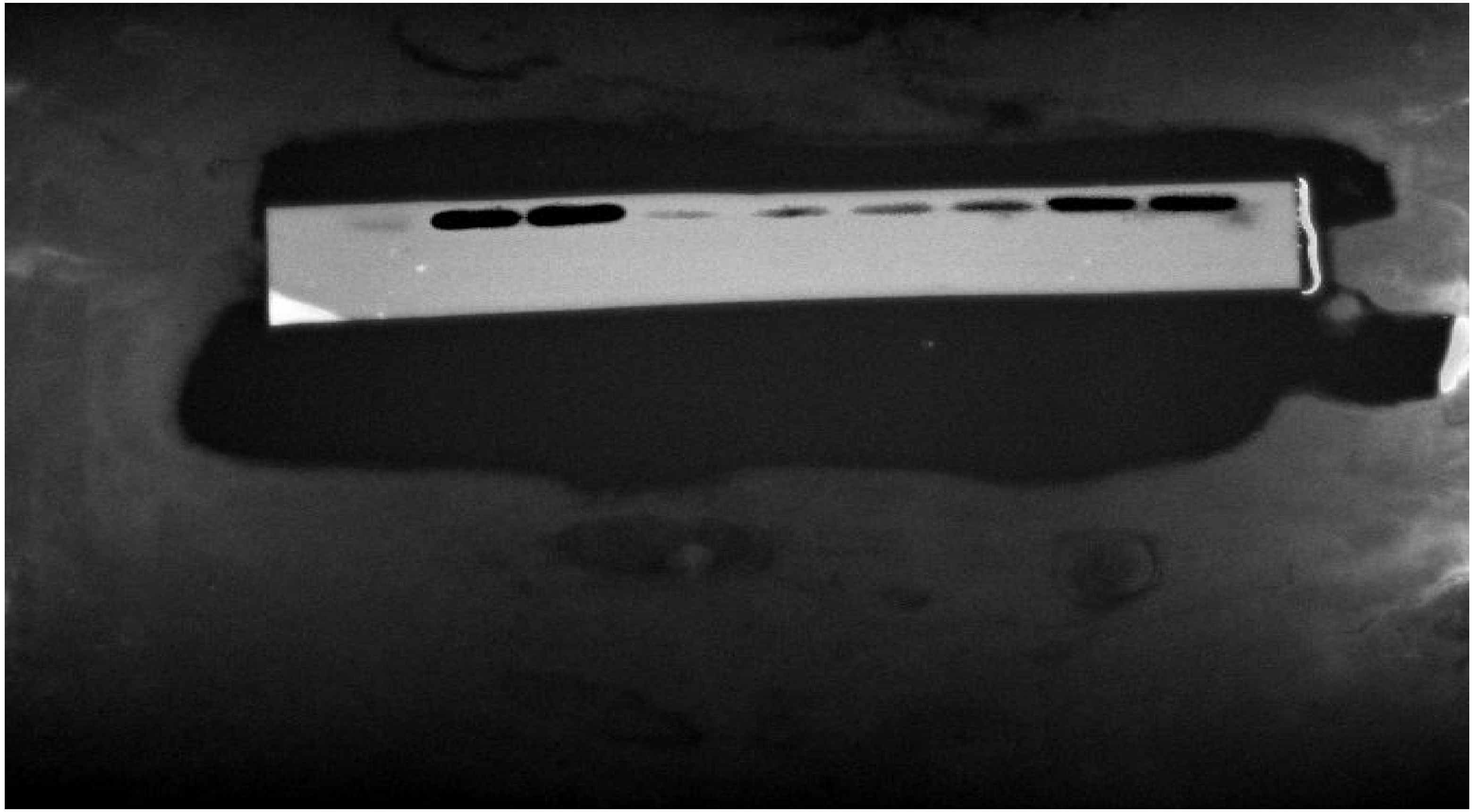

Fig9B

FTH-1

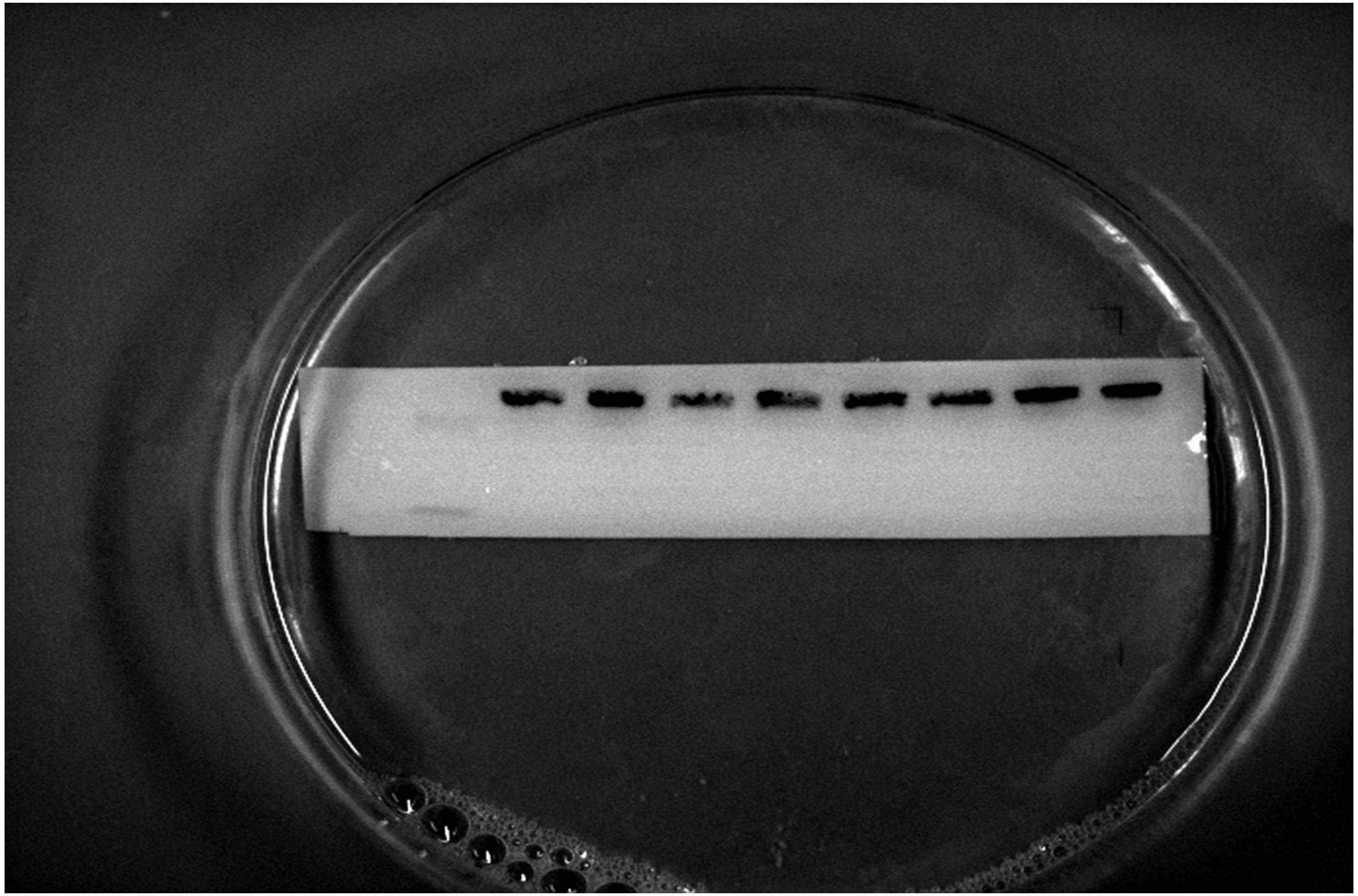

Fig9B

GPX4

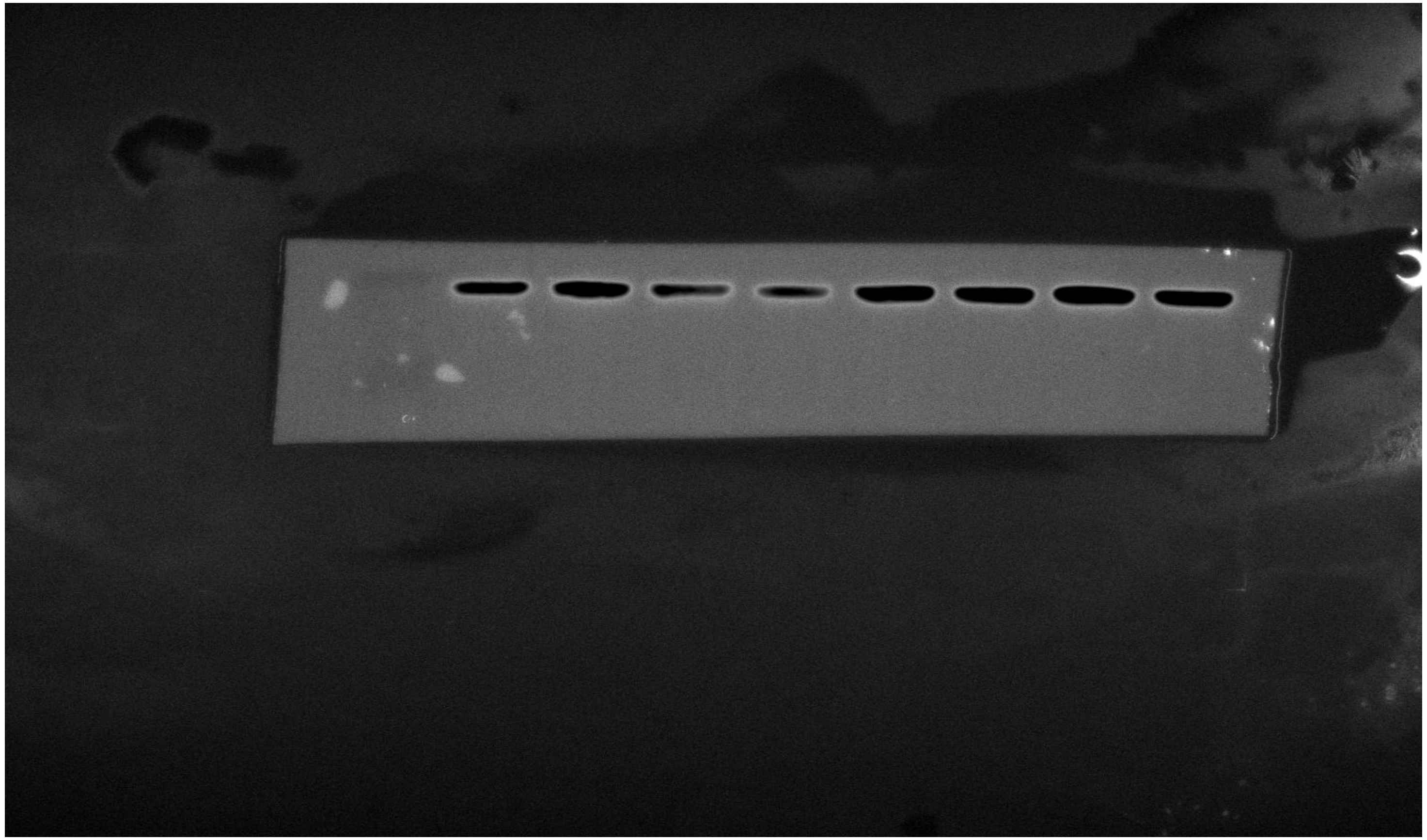

Fig9B

GAPDH

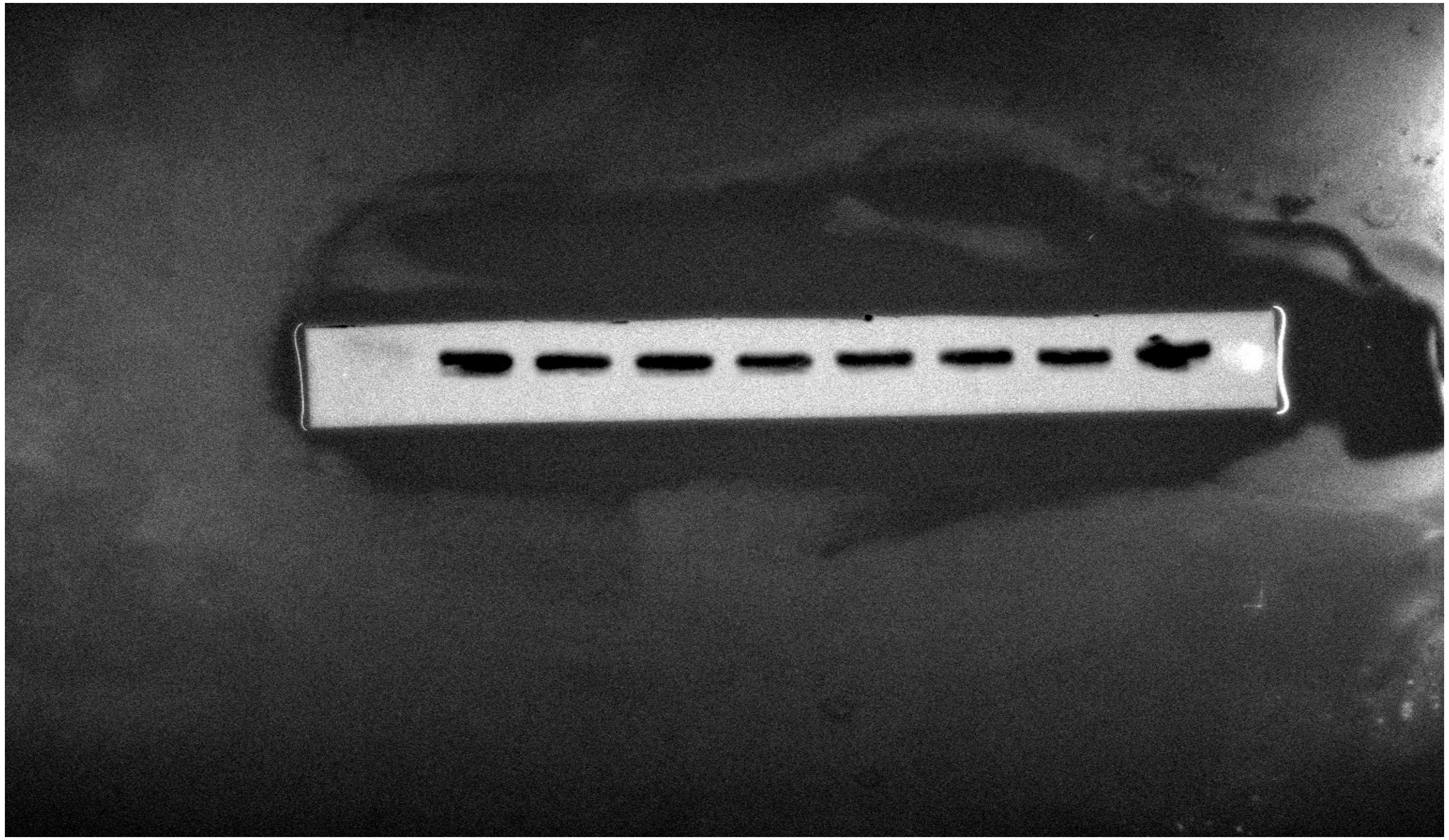

Supplement: S1 Raw images — (PDF) [file pone.0279010.s002.pdf]
