# Supplementary material for: Aspirin mediates protection from diabetic kidney disease by inducing ferroptosis inhibition
Source: PLoS One. 2022 Dec 14;17(12):e0279010. doi: 10.1371/journal.pone.0279010 (PMC9749971; doi:10.1371/journal.pone.0279010)
Supplement: Attachment — Submitted filename: Response to Reviewers.docx [file pone.0279010.s003.docx]

1、I ensure that my manuscript meets PLOS ONE's style requirements, including those for file naming.

2、 My blot/gel image data are in Supporting Information.
3、 I ensure that I have an ORCID iD and that it is validated in Editorial Manager.
4、 All mice were euthanized with 60 mg/ml of pentobarbital sodium at the end of the experimental period.
5、 I have revised the manuscript to rephrase the duplicated text.
6、 All authors have declared that no competing interests that pertain to this work.This information have be included in my cover letter.

Reviewer #1

Q1:DCHF-DA ( 2′ ,7′ -dichlorofluorescein diacetate) can be used to detect total intracellular ROS, the expression in the text has been modified by us.

Q2:The viability of cells treating with RSL-3 was assessed by CCK8. Our study showed that as RSL-3 concentration increased as it was obviously decreased . When RLS-3 concentration was over 0.1 μM, it decreased obviously. As the HK-2 cells was treated with different concentrations of RSL-3 for 48 h, the cell viability was partially recused by Fer-1 400nm or AS 400um treatment. In Fer-1 group and AS group , cells were respectively cultured in 30 mmol/L glucose medium supplemented with 400nM Fer-1 and 400 μM Aspirin for 48 h .

Q3:In this study, we mainly explored the relationship between aspirin and ferroptosis, so we did not examine other antioxidant and oxidative stress protein.

Q4:Theultrastructural analysis displayed that the changes of mitochondria morphological in the HK-2 cells cultured with high glucose, containing increased membrane density with shrunken mitochondria and mitochondrial ridge decrease or even vanishing, were alleviated by aspirin. The mitochondrial ridge remains obviously visible , the status of increased membrane density and mitochondrial shrinkage were weakened in AS group (Fig 9L). The research implied that the mitochondrial morphological pathogenesis caused by diabetic ferroptosis could be ameliorated by aspirin.

Q5:I have fixed the grammar mistakes in the article.

Reviewer #2

Q1：In this study, the end products of lipid peroxidation ,MDA,was detected in the cells and animal.

Q2：In Fig 4B, COX2 level is increased with DMSO treatment. Because cells were respectively cultured in 30 mmol/L glucose medium supplemented with 0.1% DMSO for 48 h.

Q3: In cells being cultured in high-glucose medium with Aspirin,the protein expression of SLC7A11,GPX4, FTH-1 were obviously upregulated while TFR-1 was decreased compared to cells under high-glucose medium ((Fig 4B), these genes exhibited the parallel mRNA expression (Fig 4C-4F).Aspirin treatment markedly attenuated the MDA level of HG group cell(Fig 4I). The GSH was also obviously decreased and the levels of iron was higher in HG group cells,the changes were ameliorated by aspirin in high-glucose medium (Fig 4H,4J).Moreover, the mRNA levels of KIM-1 and NGAL significantly increased in high glucose-cultured HK-2 cells,and these changes were alleviated by aspirin.(Fig 4K-4L)
