# Supplementary material for: Aspirin mediates protection from diabetic kidney disease by inducing ferroptosis inhibition
Source: PLoS One. 2022 Dec 14;17(12):e0279010. doi: 10.1371/journal.pone.0279010 (PMC9749971; doi:10.1371/journal.pone.0279010)
Supplement: Attachment — Submitted filename: Response to Reviewers.docx [file pone.0279010.s004.docx]

Dear Editor:

I would like to thank the reviewers for their careful review and valuable comments on our manuscript. Actually, we have tried our best to revise the manuscript with my co-authors. We carefully revised the manuscript according to each point of the comments. We hope that our revised version will be satisfactory for publication in *PLOS ONE*. Great thanks to you and the referee for your kind help and attention on this paper. I am looking forward to hearing from you.

Thank you very much.

Sincerely,

Zhongming Wu, Ph.D.

Reviewer #1: This article contain some typographical and grammatical errors that cannot be overlooked.

Thank you for your kind suggestion.We have described these sentence in clearer manner in the revised manuscript, as follows:

1. Abstract, line 4 and 5: Ferroptosis is a novelty the term linked to lipid hydroperoxidation, and it takes an important part in the pathogenesis of DKD.

Reply: Ferroptosis is a novel term linked to lipid hydroperoxidation, and it plays an important role in the pathogenesis of DKD.

2. "DN models" were mentioned in the manuscript multiple times but I believe it should be DM. Please clarify the meaning of DN.

Reply:Thank you for your nice advice.We have removed the DN models from the article.

3. Introduction, line 28: Ferroptosis is a novel nonapoptotic cell death that is discovered iron-dependent form in 2012, induced by a redox imbalance redox homeostasis, which is characterized by detoxify lipid oxidation product and free radicals [11].

Reply:Ferroptosis is a novel nonapoptotic cell death that was discovered to be iron-dependent in 2012 and induced by redox imbalance redox homeostasis, which is characterized by detoxification of lipid oxidation products and free radicals [11].

4. "These suggesting that by inhibiting ferroptosis in injury PTECs may become a new treatment for kidney"

Reply:These results suggest that inhibiting ferroptosis in injured PTECs may become a new treatment for kidney diseases.

5. "COX2 as an cyclooxygenase super-family protein that is an inducible enzyme at low levels in most tissues and significantly increased in the proximal tubule of the injured kidney [25,26]"

Reply:COX2 is a cyclooxygenase superfamily protein that is an inducible enzyme at low levels in most tissues and is significantly increased in the proximal tubule of the injured kidney [25,26].

6. Results, 3.1, line 22: "Simultaneously, the data showed that the end products of lipid peroxidation, Micro Malondialdehyde Assay Kit was used to assess the level of Malondialdehyde (MDA), the production of MDA was increased by 50% (Fig 1I) compared with the Ctrl group.

Reply:Simultaneously, the data showed the end products of lipid peroxidation; a micro malondialdehyde assay kit was used to assess the level of malondialdehyde (MDA), and the production of MDA was increased by 50% (Fig. 1I) compared with that in the Ctrl group.

7. "We hypothesized that RSL3 was applied to cause ferroptosis in HK-2 cells in order to further explore the role of COX2 in ferroptosis."

Reply:We utilized RSL3 to cause ferroptosis in HK-2 cells in order to further explore the role of COX2 in ferroptosis.

8. Furthermore, the viability of cells treating with RSL-3 was assessed by CCK8.

Reply:Furthermore, the viability of cells treated with RSL-3 was assessed by CCK8 assay.

9. Moreover, the lipid peroxidation product accumulation,MDA(Fig 6K) and high the concentrations of iron (Fig 6L) under ferroptosis conditions were also attenuated after Fer-1 or aspirin treatment.

Reply:Moreover, lipid peroxidation product accumulation, MDA (Fig. 6K) and concentrations of iron (Fig. 6L) under ferroptosis conditions were also attenuated after Fer-1 or aspirin treatment.

Reviewer #2: The author answered most of the questions, but the author didn't address the 4-HNE level in high glucose incubated HK2 cells.

Thank you for your kind suggestion. The products of lipid peroxidation involve reactive aldehydes (e.g. 4-hydroxynonenal (4HNE) and malondialdehyde (MDA)), which increase during ferroptosis . In this study, we have added experiments on 4-HNE levels of the cells.The products of lipid peroxidation, 4-HNE(Fig. 1J) was dramatically elevated in in HG group cells.Normally, untreated control cells contained only a minimal amount of 4-HNE. The cells treated with high glucose showed the maximal level compared to NC group cells. Interestingly, aspirin treatment markedly attenuated the level compared to that in HG group cells (Fig. 4J). Moreover,the higher levels of 4-HNE were also observed in high glucose-treated HK-2 cells, and this decrease was ameliorated in high glucose-cultured cells transfected with COX2-siRNA (Fig. 5J). The lipid peroxidation product accumulation, 4-HNE(Fig.6L) under ferroptosis induced by cells cultured in 100 nm RSL-3 medium was also attenuated after Fer-1 or aspirin treatment.Simultaneously, pretreatment with transfection of COX2-siRNA reduced the concentrations of 4-HNE（Fig.7K）, which implied that transfection of COX2-siRNA could improve the states of lipid peroxidation induced by cells cultured in 100 nm RSL-3 medium.
